# Evaluation of the diagnostic value of transcranial electrical stimulation (TES) to assess neuronal functional integrity in horses

**DOI:** 10.3389/fnins.2024.1342803

**Published:** 2024-04-11

**Authors:** Sanne Lotte Journée, Henricus Louis Journée, Wilhelmina Bergmann, Ilias Chantziaras, Katrien Vanderperren, Els Raes, Stephen Michael Reed, Cornelis Marinus de Bruijn, Hanneke Irene Berends, Cathérine John Ghislaine Delesalle

**Affiliations:** ^1^Equine Diagnostics, Wijns, Netherlands; ^2^Department of Translational Physiology, Infectiology and Public Health, Research Group of Comparative Physiology, Faculty of Veterinary Medicine, Ghent University, Merelbeke, Belgium; ^3^Department of Neurosurgery, University Medical Center Groningen, University of Groningen, Groningen, Netherlands; ^4^Department of Orthopedics, University Medical Center Utrecht, Utrecht University, Utrecht, Netherlands; ^5^Department of Orthopedics, Amsterdam University Medical Center, Amsterdam, Netherlands; ^6^Department of Biomolecular Health Sciences, Division of Pathology, Faculty of Veterinary Medicine, Utrecht University, Utrecht, Netherlands; ^7^Department of Internal Medicine, Reproduction and Population Medicine, Faculty of Veterinary Medicine, Ghent University, Merelbeke, Belgium; ^8^Department of Morphology, Imaging, Orthopedics, Rehabilitation and Nutrition, Faculty of Veterinary Medicine, Ghent University, Merelbeke, Belgium; ^9^Rood and Riddle Equine Hospital, Lexington, KY, United States; ^10^Maxwell H. Gluck Equine Research Center, Department of Veterinary Science, University of Kentucky, Lexington, KY, United States; ^11^Wolvega Equine Clinic, Oldeholtpade, Netherlands

**Keywords:** horses, ataxia, transcranial stimulation, myelography, neurology, necropsy, motor potentials

## Abstract

Medical imaging allows for the visualization of spinal cord compression sites; however, it is impossible to assess the impact of visible stenotic sites on neuronal functioning, which is crucial information to formulate a correct prognosis and install targeted therapy. It is hypothesized that with the transcranial electrical stimulation (TES) technique, neurological impairment can be reliably diagnosed.

**Objective:**

To evaluate the ability of the TES technique to assess neuronal functional integrity in ataxic horses by recording TES-induced muscular evoked potentials (MEPs) in three different muscles and to structurally involve multiple ancillary diagnostic techniques, such as clinical neurological examination, plain radiography (RX) with ratio assessment, contrast myelography, and post-mortem gross and histopathological examination.

**Methods:**

Nine ataxic horses, showing combined fore and hindlimb ataxia (grades 2–4), were involved, together with 12 healthy horses. TES-induced MEPs were recorded bilaterally at the level of the trapezius (TR), the extensor carpi radialis (ECR), and tibialis cranialis (TC) muscles. Two Board-certified radiologists evaluated intra- and inter-sagittal diameter ratios on RX, reductions of dorsal contrast columns, and dural diameters (range skull-T1). Post-mortem gross pathological and segmental histopathological examination was also performed by a Board-certified pathologist.

**Results:**

TES-MEP latencies were significantly prolonged in both ECR and TC in all ataxic horses as opposed to the healthy horses. The TR showed a mixed pattern of normal and prolonged latency times. TES-MEP amplitudes were the least discriminative between healthy and ataxic horses. Youden’s cutoff latencies for ataxic horses were 24.6 ms for the ECR and 45.5 ms for the TC (sensitivity and specificity of 100%). For healthy horses, maximum latency values were 22 and 37 ms, respectively. RX revealed spinal cord compression in 8 out of 9 involved ataxic horses with positive predictive values of 0–100%. All ataxic horses showed multi-segmental Wallerian degeneration. All pathological changes recorded in the white matter of the spinal cord were widely dispersed across all cervical segments, whereas gray matter damage was more localized at the specific segmental level.

**Conclusion:**

TES-MEP latencies are highly sensitive to detect impairment of spinal cord motor functions for mild-to-severe ataxia (grades 2–4).

## Introduction

Ataxia is a commonly encountered symptom in horses and can be triggered by a variety of causes ranging from infectious and toxicological to skeletal causes, trauma, and cervical vertebral malformation. By far, the most common cause of ataxia in horses is spinal cord compression. The symptomatology can be very subtle and challenging to identify. Moreover, the sites of compression along the course of the spinal canal that appear on diagnostic imaging or contrast myelography cannot always be linked one-on-one to a functional impact. Moreover, diagnosing ataxia can be even more challenging when spinal cord compressions are dynamic and not visible during diagnostic imaging (Janes et al., 2014; Sleutjens et al., 2014; Lindgren et al., 2020). In older competition horses, wear and tear lesions can manifest themselves simultaneously at the level of many spinal locations. In such cases, it is a real challenge for a clinician to determine which lesions have actual neuronal impact. The aforementioned clearly emphasizes the need for the development and optimization of neurophysiological techniques that actually assess neuronal functional integrity. Such techniques are important not only to formulate a proper and scientifically based prognosis but also to allow for objective follow-up of the progression of neurological patients throughout treatment and rehabilitation.

To study the value of transcranial electrical stimulation (TES) as a diagnostic tool, it was decided to focus on the most common causative pathology of spinal ataxia in horses, in particular, cervical vertebral compressive myelopathy (CVCM) (Levine et al., 2007; Van Biervliet, 2007). CVCM is the result of compression of the cervical spinal cord and/or spinal nerves. This compression may be either dynamic (only apparent in flexed head and neck positions) or static (always present). Stenoses can cause functional neuronal perturbation and, in a second step, neuronal tissue damage in the spinal cord. In addition, peri-articular proliferation invading the inter-vertebral foramina can cause cervical nerve root compression, neck pain, and forelimb lameness (Ricardi and Dyson, 1993; Marks, 1999).

A wide array of different medical imaging techniques can be employed during the diagnostic work-up of the neurological patient. Although many of these medical imaging techniques have undergone enormous evolution in recent decades, the spinal canal of the horse can still be difficult to evaluate in detail because of either the occurrence of superposition of anatomical structures or simply the massive body volume of the horse hampering the achievement of high-quality images (Garrett, 2022). Plain radiographs provide a static view of the spinal canal. Instead of a “subjective” assessment of the obtained images, the determination and calculation of intra- and inter-vertebral sagittal diameters are advocated (Van Biervliet, 2007; Bedenice and Johnson, 2022). Additionally, to further outline the spinal cord, contrast myelography can be performed in neutral, flexed, and extended neck positions (Levine et al., 2007, 2010; Scrivani et al., 2011; Hughes et al., 2014). Ultrasound can be useful in detecting osteochondrosis of cervical joints, but its diagnostic validity is subject to debate (Garrett, 2022; Hoey et al., 2022). Computed tomography (CT) and magnetic resonance imaging (MRI) can provide a view of the spinal canal in all directions (Lindgren et al., 2020; Rovel et al., 2021) and greatly improve the clinical evaluation of CVCM in horses (Janes et al., 2014; Sleutjens et al., 2014; Lindgren et al., 2020). However, it needs to be emphasized that none of the medical imaging techniques provides objective information about the neuronal functional integrity of the patient.

Post-mortem gross pathological examination combined with detailed segmental histopathological examination is often considered the “gold standard” for identifying neuronal tissue damage because it provides a detailed and direct visualization of the tissue at the anatomical, cellular, and structural levels (Summers et al., 1995). Therefore, this approach was chosen as “golden standard” in the current study. Gross pathology combined with histopathological evaluation offers a solid approach to underpin TES findings, allowing for the assessment of the sensitivity and specificity of this neurophysiological technique. Throughout gross pathology, the focus is directed toward identifying the possible presence of discoloration, necrosis, hemorrhages, and more specially for CVCM space-occupying lesions and areas of compression at the level of the spinal cord and/or spinal nerves. Segmental histopathology comprises the evaluation of the gray and white matter, blood vessels, meninges, and the central canal for signs of inflammation, vascular changes, necrosis, degeneration, and neoplasia. For CVCM, the site of compression can be determined by the presence of Wallerian degeneration (swollen axons/spheroids, dilated myelin sheaths, and digestion chambers with macrophages) randomly distributed in all funiculi. At the site of compression, degeneration, necrosis, and loss of motor neurons may also be seen in the gray matter. Cranial to the site of compression, Wallerian degeneration is visible in the ascending tracts and caudal to the site of compression in the descending tracts. In longstanding chronic cases, sclerosis may be visible, replacing the normal nervous tissue (Summers et al., 1995). Wallerian degeneration is associated with the slowing down or blocking of axonal conduction. It is a process of neuronal degeneration associated with damaged myelin layers of axons spread across one or multiple spinal segments, usually due to injury or damage. The deceleration of axonal conduction and the loss of motor axon conduction are expected to result in increased measured muscular evoked potential (MEP) latencies and a decrease in the amplitudes.

Indeed, Wallerian degeneration may also be visible in the spinal nerves. In chronic cases, histological changes consistent with chronic compression neuropathy (epineural and perineural fibrosis, increased numbers of Renaut bodies, Schwann cell proliferation, and thickening of myelin sheaths and remyelinating fibers) may be seen in nerves stained with toluidine blue (Duncan et al., 1987). In addition to spinal compression, generalized neurotrophic diseases can induce ataxia in horses. Vitamin E deficiencies, for example, can lead to impairment of neuromuscular functions, resulting in ataxia and muscle atrophy. In rats, electrophysiological studies have shown that muscle degeneration precedes degeneration of peripheral nerves (Einarson, 1953; Pillai et al., 1994). Necrosis of type I muscle fibers occurs along with increased spheroid formation in the gracilis and cuneate nuclei of the brainstem. The histologic lesion associated with neuroaxial dystrophy/equine degenerative myeloencephalopathy (EDM) is central axonal degeneration, which is most pronounced in the somatosensory tracts (spinocuneo-cerebellar and dorsal spinocerebellar tract), whereas lesions associated with equine motor neuron disease (EMND) include chromatolysis of neurons within the ventral columns as well as peripheral axonal degeneration and associated neurogenic atrophy of muscle fibers (Finno et al., 2016).

To achieve a proper evaluation of the TES technique to reliably determine the neuronal functional integrity in an equine neurological patient, additional diagnostic techniques were also included in the current study, such as an appropriate clinical neurological examination, plain radiography (RX), contrast myelography, and post-mortem gross pathology and detailed segmental histopathology. To the best of our knowledge, a check of obtained transcranial generated muscle potential parameters to assess functional performance of the spinal cord in horses, against the results of ancillary diagnostic techniques such as medical imaging, is only described for TMS and in a single study for a limited number of included horses in which, in addition to clinical neurological evaluation, plain radiography, myelograms and post-mortem examinations were performed.

It is hypothesized that with the TES technique, functional neurological impairment can be reliably diagnosed and that this neurophysiological technique is better suited to achieve that goal when compared to medical imaging techniques.

To prove the hypothesis, TES-induced MEPs were recorded bilaterally in three different muscles [m. extensor carpi radialis (ECR), m. tibialis cranialis (TC), and m. trapezius (TR)] and a multitude of ancillary diagnostic approaches were included, such as clinical neurological examination, plain radiography with ratio assessment, contrast myelography, and post-mortem examination (both gross pathology and segmental histopathology). Histopathology was used as the golden standard to assess the presence of neuronal tissue damage.

## Materials and methods

Horses suspected of suffering from cervical spinal ataxia, of which horse owners opted for humane euthanasia after diagnosis, were involved. Only horses showing both fore and hindlimb ataxia were included. The ataxic group consisted of 9 horses: 4 mares, 4 stallions, and 1 gelding, aged between 0.9 and 11.7 (3.4 ± 3.3; mean ± SD) years, body weight ranging from 330 to 618 (497 ± 96) kg, and height at withers ranging from 147 to 163 (156 ± 4.6) cm. The following breeds were involved: 4 Dutch warmblood horses, 1 standardbred, 1 Friesian, and 3 Groninger horses, which were all referred to the Wolvega Equine Clinic, the Netherlands.

A control group of 12 horses was included from a previously published prospective study (Journée et al., 2018) and consisted of 6 mares and 6 geldings, aged between 3.6 and 20.53 (10.7 ± 5.5) years, body weight 448–749 (589 ± 101) kg, and height at withers 138–178 (161 ± 10) cm, including 2 Standardbred, 2 Friesian, 1 Rheinlander, 1 Oldenburger, 1 Tinker, and 5 Dutch warmblood horses.

### Neurological examination

Before sedation, each horse underwent a clinical neurological examination by a European Board-certified equine veterinarian (ECEIM) (MdB). The severity of signs was scored according to the grading scale of Mayhew et al. (1978). This scale ranges in severity from 0 to 5, in which 0 indicates the absence of neurological signs while 5 indicates recumbency.

### TES procedure

The TES procedure was performed as previously described (Journée et al., 2018). In brief, the neurophysiological recordings were performed with a multichannel neurophysiological monitoring system (Neuro-Guard JS Center, Bedum, The Netherlands). TES was performed using biphasic constant voltage stimulation involving three pulses per train and an interpulse interval of 1.3 ms, applying incremental voltage series as previously described (Journée et al., 2018).

The MEPs were recorded simultaneously and bilaterally from the trapezius (TR), the extensor carpi radialis (ECR), and the tibialis cranialis muscles (TC). Subcutaneous needle electrodes (82015-PT L 12 mm 27GA Rochester Lutz, FL, United States) were inserted in the TR [20 cm separated, at 60 cm distance from the protuberance of the skull (inion)], ECR (10 and 20 cm above the os carpi accessorium), and the TC (10 and 20 cm above the medial malleolus). A ground needle electrode was placed subcutaneously in the neck.

The signals were band-filtered between 50 and 2,500 Hz (3 dB) and digitally stored for post-hoc processing. Latencies and amplitudes of the early MEP components in the transcranial time window were obtained at stimulation intensities around 20 V above the transcranial threshold.

### Diagnostic imaging of ataxic horses

Standing latero-lateral radiographs were taken in a neutral position after sedation with 0.012 mcg/kg body weight (bwt) detomidine (Detosedan, AST Farma B.V., Oudewater, The Netherlands) and 0.025 mcg/kg bwt butorphanol (Butomidor AST Farma B.V., Oudewater, The Netherlands). After the radiographs were taken, the horses were prepared for myelography. They were induced with 0.06 mcg/kg bwt midazolam (Dormazolam Dechra, Bladel, The Netherlands) and 2.2 mcg/kg bwt ketamine (Ketamine AST Farma B.V., Oudewater, The Netherlands). General anesthesia was maintained using continuous rate infusion with a triple drip of 250 mg guaifenesin 10% (Gujatal Dechra, Bladel, The Netherlands), 2.25 g sodium chloride 0.9% (saline solution for infusion, Dechra, Bladel, The Netherlands), 2 g ketamine 10% (AST Farma B.V., Oudewater), and 25 mg romifidine 1% (Rominervin AST Farma B.V., Oudewater). The atlanto-occipital area was surgically prepared, and after the removal of 50 mL cerebrospinal fluid, 50 mL of undiluted iohexol (Omnipaque 300 mg I/ml, GE Healthcare B.V., Eindhoven, The Netherlands) was injected slowly in the epidural space using an 18G spinal needle. Latero-lateral radiographs were taken with the horse in lateral decubitus in three neck positions: neutral, flexed, and extended.

The intra- and inter-vertebral sagittal diameter ratios of the plain cervical radiographs of the vertebral canal were measured at each cervical vertebral junction starting from the skull all the way to T1 as described by Hahn et al. (2008). To distinguish between a normal and a narrowed vertebral canal for both ratios, a cutoff value of 0.485 was used.

In cervical myelograms, measurements of the dorsal contrast column and sagittal dural diameter were performed at the level of the inter-vertebral articulation between all cervical vertebrae and at the mid aspect of each vertebra. A 50% reduction of the dorsal contrast column and a reduction of 20% of the sagittal dural diameter was considered as significant (Nout and Reed, 2003), while for the cervicothoracic junction, a 60% reduction was considered as significant (Estell et al., 2018). Three neck positions were assessed: neutral, flexed, and extended. Radiographic measurements were performed independently, on each occasion, by two medical imaging Board-certified specialists (KvP and ER). Both assessors were blinded to the clinical status of the involved horses.

### Gross pathology and histopathology

All horses that were referred to the clinic with a poor prognosis and of which horse owners opted for euthanasia were euthanized and subsequently subjected to gross pathology and segmental histopathology.

Horses underwent a full necropsy. Subsequently, the left and right spinal nerves from C1 up to C8, together with a small amount of surrounding musculature, were sampled. Then, the neck and head were removed from the rest of the body by sawing through the third thoracic vertebra. After removing the musculature surrounding the neck, the processes of the spinalis were removed by sawing through the vertebral laminae. The cervical spine, including the removed laminae, was then examined for space-occupying lesions and narrowing of the vertebral canal, referred to as “stenosis.” This was done with the spine in neutral, extended, and flexed positions. Next, the spinal cord was removed, and the spinal canal was again examined for the presence of space-occupying lesions in the three different positions. Hereafter, the spinal canal was sawed longitudinally in half, after which examination for space-occupying lesions was repeated. The caudal articular process joints of the second cervical vertebrae up to the cranial articular process joint of the first thoracic vertebra were examined. The macroscopic presence or absence of stenoses was scored by the pathologist at the specific anatomical segment (range SK-T1).

Across the full length of the cervical region (SK-T1), the sampled musculature, nerves, and spinal cord were fixed in 10% buffered formalin and, after fixation, routinely processed, paraffin-embedded, stained with hematoxylin and eosin, and evaluated by light microscopy by a Board-certified veterinary pathologist (WB).

If histopathological signs of compression were visible, these were scored at the specific segmental location (Table 1). The severity of lesions within the white and gray matter of the spinal cord was scored as either mild, moderate, or severe, and the segmental location at which they were encountered was indicated (Table 1). Lesions within segmental nerves and denervation atrophy of segmental muscles were per body side (left vs. right) scored as either mild, moderate, or severe (Table 1). When the pathological changes of the nervous tissue were consistent with CVCM, the site of compression was also determined by separate evaluation of the ascending and descending white matter tracts in the spinal cord (Summers et al., 1995).

**Table 1:** Overview of how applied histopathological gradings were defined throughout all assessed parameters across patient samples for, respectively, changes detected in the gray matter of the spinal cord, signs of Wallerian degeneration, signs of nerve compression, and signs of muscular atrophy due to denervation.

Histopathological gradings of lesions in the grey and white matter of the spinal cord, spinal nerves and denervation atrophy of the muscles	
Changes Gray matter in spinal cord	
Mild	Mild increase (up to 25%) in glia cells (gliosis)
Moderate	Moderate gliosis (increase ranging from 25 to 50%)
Severe	Moderate to severe (increase equal to or above 50%) gliosis, neuronal necrosis, neuronal loss, and or glia nodules
Severity of Wallerian degneration	
Mild	Presence of 1–5 spheroids and/or digestion chambers in at least one of 10 high power fields (400x)
Moderate	Presence of 5–20 speroids and/or digestion chambers in at least one of 10 high power fields (400x)
Severe	20 or more speroids and/or digestion chambers in at least one of 10 high power fields
Severity of changes associated with nerve compression	
Mild	Presence of mild perineural fibrosis
Moderate	Combined presence of mild to moderate perineural fibrosis; presence of epineural fibrosis; increased amounts of Renaut bodies
Severe	Combined presence of moderate to severe perineural fibrosis, presence of epineural fibrosis, increased amounts of Renaut bodies, formation of Bungerbands, presence of inflammation
Severity of changes associated with musle denervation atrophy	
Mild	Absence of one or more small groups of atrophied muscle fibers per high powerfield
Moderate	Detection of one or more small groups of atrophied muscle fibres in at least one of 10 high power fields
Severe	Presence of extensive denervation atrophy

### Statistical analysis

Statistical analysis was performed with SPSS™ software, version 20.0.0, IBM™_._

### Assessment of TES-induced MEP parameters

For both groups, the MEP latency times were assessed separately for the TR, ECR, and TC. The latencies of the healthy horse group were computed as mean latencies at suprathreshold TES intensities for each case, as previously described (Journée et al., 2018).

Latencies of the ataxic group were selected from the lowest of two reproducible values above the intracranial TES intensity threshold. Box plots of TES-MEP latencies and amplitudes of the ECR and TC as a function of the ataxia grading (as an ordinal variable) were composed with the computation of regression lines and Spearman’s correlations. The histograms and receiver operating curves (ROCs) include latencies from the left and right sides and are intended to differentiate between an ataxic horse and the healthy horse group. The Youden’s index (sensitivity + specificity −1) was calculated to determine optimal cutoff values for latency times. YC indicates the optimum cutoff of the latency where Youden’s index is maximal. The cutoff values for the ECR and TC muscles are indicated by YC_ECR_ and YC_TC_, respectively. An additional latency cutoff to discriminate between normal and pathological prolonged latencies is defined at the 95% confidence interval limit of the healthy horse group. For the calculation of the upper 95% limit of normal MEP latency values in healthy horses, it is assumed that these are normally distributed.

Normal 95% latency limits for the TR, ECR, and TC muscles are indicated by NL_TR_, NL_ECR_, and NL_TC_, respectively.

### Processing and visualization of radiological scores and necropsy parameters

The obtained TES-MEP latencies and amplitudes are represented, together with the radiological and histopathological findings in the results section according to increasing classes of ataxia scores in Table 2.

**Table 2:** Overview of patient characteristics and findings per horse.

Horse	Sex	Breed	Age y	Description	Ataxia grade	Prolonged latency			Stenoses medical imaging		Stenoses		Pathology					
													Location and severity scores		Seg-mental nerve		Seg-mental muscle	
						TR	EC	TC	RX	MY	Mac	Mic	White matter	Grey matter	Left	Right	Left	Right
A	m	Dutch warmblood	2.6	Ataxia pelvic>thoracic limbs dynamic wobbler C3/4 & C4/C5 no visible denervation atrophies	2	**X**	**X**	**X**					Sk-C1 C1/2 C2/3 C4/5 C5/6 C6/7 C7-T1	C6/7 C7-T1		**C8**	C6	
B	st	Dutch warmblood	4.3	Ataxia in thoracal and pelvic limbs dynamic cervical myelopathy PA: skull to C4 likely location denervation atrophy	3	**X**	**X**	**X**	C6/7	C2/3		Sk-C1 C1/2	**Sk-C1** C1/2 C2/3 C7T1	C7T1	**C3** **C4**	**C3**	C4	**C5** **C7**
C	st	Groninger	2.3	Ataxia pelvic=thoracic limbs cervical dynamic myelopathy PA primary cause skull-C2 stenosis denervation signs in all muscles	3	–	**X**	**x**	C5/6	C4/5	C3/4	C2/3 C3/4	Sk-C1 **C1/2** **C2/3** **C3/4** **C4/5** **C5/6** **C6/7**	C5/6	**C3** **C4** **C5** **C7**	**C2** **C3** **C4** **C5** **C6** **C7** **C8**	C4	**C4**
D	m	Friesian	1.0	Ataxia pelvic>thoracic limbs cervical myelopathy static myelopathy denervation signs muscles	3	**–**	**X**	**X**		C1/2 C3/4 C4/5 **C6/7**	C6/7	C5/6	**Sk-C1** **C1/2** **C2/3** **C3/4** **C4/5** **C5/6** **C6/7** **C7T1**	Sk-C1 C1/2 **C2/3** C3/4 C4/5 **C5/6** C7T1	C3 C4 C6 C7	**C8**		
E	g	Standard-bred	3.0	RX and myelographic stenosis static myelopathy denervation signs muscles	3	**X**	**X**	**X**	**C5/6** **C6/7**	C1/2 C3/4 C4/5 C6/7		Sk-C1	**Sk-C1** **C1/2** **C2/3** **C3/4** **C4/5** C5/6 C6/7 **C7-T1**	Sk-C1 C1/2 C3/4 C4/5	C3 **C4** C8	C3		**C7**
F	st	Groninger	2.2	Ataxia PA no stenoses + grey matter degeneration and Wallerian degeneration in brain stem ➔ suspected EDM mild signs of denervation & muscular atrophy	4	–	**X**	**X**	**C3/4** **C4/5**	C1/2 C3/4 **C5/6** **C6/7**			**Brain stem** **Sk-C1** **C1/2** **C2/3** **C3/4** **C4/5** **C5/6** **C6/7** **C7-T1** **T?/?**	**Brain stem** **T?/?**	**C3** **C5** **C7** C8	C3 C4 C5 C6 C7	C7	**C7** **C8**
G	st	Groninger	1.7	Cervical dynamic myelopathy mild denervation and muscular atrophy	4	–	**X**	**X**	C2/3 C3/4	C2/3 **C3/4** **C4/5**	C4/5	C3/4 C4/5	**Sk-C1** **C1/2** **C2/3** **C3/4** **C4/5** **C5/6** **C6/7** **C7T1**	**C4/5**	**C1** **C3**	C1 **C3** **C4** **C7**	C1 C3	C1 C3 **C6**
H	m	Dutch warmblood	1.7	External inspection: atrophy left serratus at alternating segments radiologic and also PA stenoses mainly at C5/6, C6/7; signs of muscular denervation in m. serratus	4	–	**X**	**X**	C3/4 C4/5 C5/6 **C6/7**	C1/2 C3/4 **C5/6** **C6/7**	C3/4 C4/5 C5/6 C6/7 C7T1	C4/5 C5/6 C6/7	Sk-C1 C1/2 C2/3 C3/4 **C4/5** **C5/6** **C6/7** C7T1	**C1/2** **C2/3** C3/4 **C4/5** **C5/6** **C6/7** **C7T1**	C1	**C2**	**C6** **C7** **C8**	C3 **C7**
I	m	Dutch warmblood	12	Spinal ataxia pelvic>thoracic limbs major osteochondrosis C6/7 denervation atrophy in many segmental nerves.	4	–	**X**	**X**	C5/6 **C6/7**	**C6/7**	C6/7	C6/7	**C1/2** **C2/3** **C3/4** **C4/5** **C5/6** **C6/7** **C7T1**	**C4/5** **C6/7** C7T1	C3 **C7**	**C2** **C3** C6 C7 **C8**		**C2**

Columns from left to right: Horse A → I; Sex: st, stallion; g, gelding; m, mare; age in years; description providing highlights concerning patient work-up; Degree of ataxia (1 → 4); Presence or absence of a prolonged TES induced MEP latency time in, respectively, the trapezius (TR), extensor carpi radialis (EC), and tibialis cranialis (TC) muscles. The characters X and x (only in TC of horse C because of absent right-sided MEP) denote bilateral and unilateral presence of prolonged MEP latencies; presence of signs of stenosis on medical imaging [Radiography (RX) versus myelography (MY)] with the corresponding anatomical location (sk-T1) bold: agreed by 2 radiological certified observers; presence or absence of signs of stenosis during gross pathology (mac) and histopathology (mic) with the corresponding anatomical location (sk-T1); histopathological assessment of the white and gray matter of the spinal cord, signs of segmental nerve compression and signs of segmental muscle atrophy due to denervation are classified as 1, 2, and 3 as defined in Table 1 and depicted as normal, bold, or underlined bold printed segmental locations, respectively. T?/?, thoracic vertebral junction at unknown segmental location.

The radiological scores of the ataxic group were coded as “positive” when at least one score per spinal segment fulfilled the described detection criteria. The aforementioned applies to the inter-and intra-segmental diameter ratios for radiographs and to the dorsal column and segmental diameter reductions visualized during contrast myelography in any of the three neck positions (normal–flexed–extended).

For each horse, the outcome of radiological evaluation and myelography were independently assessed by two Board-certified medical imaging specialists, blinded to the condition of the respective horses. Differences between scores of observers were processed for inter-observer reliability.

The inter-observer reproducibility for intra- and inter-diameter ratio assessment and dorsal column and spinal cord diameter reduction was computed as percentages of matching test scores against the total number. Intra-observer reliability was not assessed since evaluation scores were obtained only once.

## Results

The TES procedure and myelography were uneventful in all horses. All harvested CSF samples were clear/translucent without the presence of blood. Table 2 provides a summary overview of the obtained data for all ataxic horses. These concern for each horse per cervical segmental level, the radiological and myelographic findings, the post-mortem findings obtained during macroscopic and microscopic evaluation, and the histopathological scoring of the neuronal tissue damage, together with muscular atrophy assessment. The horses were grouped into ataxia scores 2 (1 horse), 3 (4 horses), and 4 (4 horses).

In addition, Table 3 provides an overview of the scorings of the histopathological changes detected for muscle atrophy in the trapezius (TR), extensor carpi radialis (EC), and tibialis cranialis (TC) muscles at the left and right body side of the involved horses.

**Table 3:** Overview of the scoring of the histopathological changes detected for muscle atrophy in, respectively, the trapezius (TR), extensor carpi radialis (ECR), and tibialis cranialis (TC) muscles at the left and right body side for Horse A → I, corresponding with the horse labels in Table 2.

Horse	Gender	Breed	Age y	Ataxia grade	Pathology scoring histopathological changes					
					TR		ECR		TC	
					Left	Right	Left	Right	Left	Right
A	m	Dutch warmblood	2.6	2	2	2	2	1	1	1
B	st	Dutch warmblood	4.3	3	2	0	0	0	2	0
C	st	Groninger	2.3	3	0	0	0	0	1	1
D	m	Friesian	1.0	3	1	0	0	1	0	1
E	g	Standardbred	3.0	3	1	1	1	0	0	0
F	st	Groninger	2.2	4	0	0	0	0	0	3
G	st	Groninger	1.7	4	0	0	0	0	0	0
H	m	Dutch warmblood	1.7	4	0	0	0	0	0	0
I	m	Dutch warmblood	12	4	0	0	0	0	0	0

### TES-MEP latencies and amplitudes versus ataxia grading

As shown in Table 4, all ataxic horses showed significantly prolonged latency times well above NL_ECR_ and NL_TC_ 95% cutoff limits determined for the healthy horse group, while the trapezius muscle showed a mixed pattern of normal and prolonged latency times, with normal latencies being detected in 6 out of 9 ataxic horses.

**Table 4:** Overview of the TES-MEP latencies for the trapezius (TR), extensor carpi radialis (ECR), and tibialis cranialis (TC) muscles at the left and right body side for horses A – I.

Horse	MEP latencies (ms)						MEP amplitudes (mV)					
	TR		ECR		TC		TR		ECR		TC	
	Left	Right	Left	Right	Left	Right	Left	Right	Left	Right	Left	Right
	NL_TR_ = 16 8.6%		NL_ECR_ = 22 6.9%		NL_TC_ = 37 7.8%		76%		69%		77%	
A	**23**	**22**	**36**	**33**	**53**	**58**	2.8	1.1	0.90	1.2	2.5	0.80
B	**34**	**35**	**32**	**29**	**93**	**85**	0.40	0.71	0.55	0.78	0.14	0.21
C	12	12	**42**	**41**	**93**		0.48	0.59	0.18	0.11	0.55	
D	**23**	**24**	**33**	**34**	**63**	**65**	4.2	4.6	1.9	2.0	0.75	0.03
E	11	11	**28**	**29**	**80**	**81**	4.6	2.4	0.71	0.56	1.3	1.6
F	11	11	**35**	**38**	**110**	**95**	2.2	1.4	0.17	0.30	0.15	0.03
G	13	12	**35**	**35**	**106**	**108**	0.56	0.21	0.61	0.48	0.15	0.21
H	15	15	**29**	**27**	**88**	**83**	2.2	1.4	2.0	0.85	0.50	0.69
I	12	12	**30**	**29**	**64**	**80**	2.6	2.5	0.52	0.60	0.43	0.46
Mean values ataxic group												
m	17	17	33	33	83	82	2.2	1.6	0.84	0.76	0.72	0.50
SD	7.8	8.3	4.1	4.6	20.0	15.6	1.5	1.3	0.68	0.55	0.76	0.51
CV	46%	48%	12%	14%	24%	19%	70%	81%	81%	73%	106%	109%
Mean of differences between sides												
m_L-R_	−0.25		0.38		0.05		0.57		0.086		0.22	
m_L-R_/m	−1.4%		1.2%		0.1%		30%		10%		31%	
SD_L-R_	0.68		1.92		9.38		0.88		0.44		0.64	

The labels correspond with those in Table 2. NL_TR_, NL_ECR_, and NL_TC,_ upper 95% limit cutoff of the latencies of the TR, ECR, and TC of the normal horse group. Prolonged latencies are printed in bold. m, mean; SD, standard deviation; CV, coefficient of variation; m_L-R_, mean of side differences; SD_L-R_, standard deviation of the mean of side differences.

There were no significant differences between latency times when comparing the left with the right body side. In the group of ataxic horses, the coefficients of variation (CVs) of the mean latency times per muscle group side are higher for the latency times of the TR: 46%; 48% (left; right) when compared to the ECR: 12%; 14% and the TC 24%; 19%. The higher CVs found for the TC compared to the ECR are explained by the dispersion of the latencies over a larger range of 53–110 ms versus 27–42 ms.

**Figure 1:**
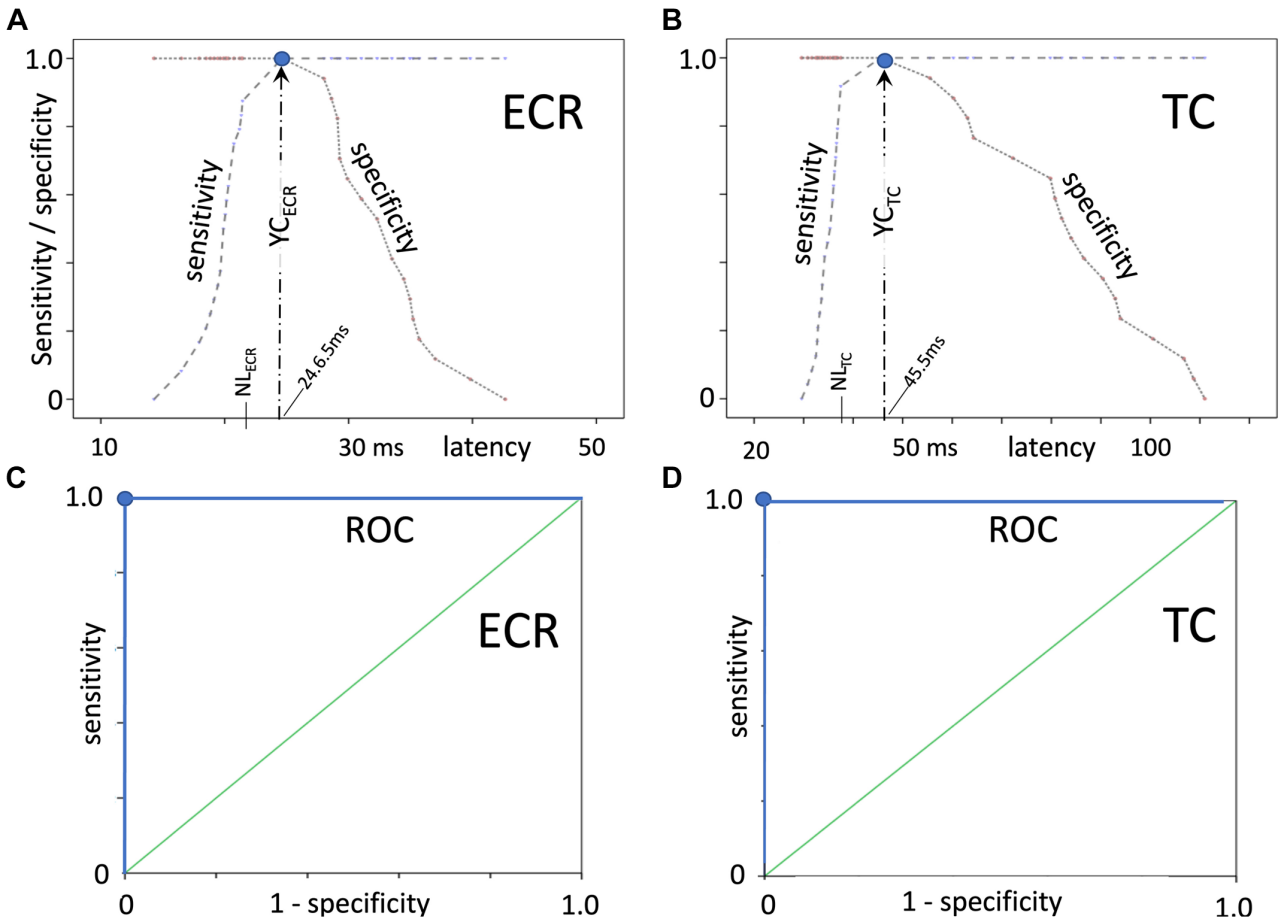
Sensitivity/specificity curves as a function of the latency, with **(A)** illustrating the Youden’s index YC_ECR_ for the ECR and **(B)** the Youden’s index YC_TC_ for the TC muscle groups. Panels **(C,D)** depict the corresponding separate receiver operating characteristic (ROC) graphs for TES-MEP latencies from these muscle groups on both body sides (combined left and right body sides) in all horses. YC indicates the optimum cutoff of the latency where both the sensitivity and specificity are equal to 1 (denoted as a blue dot in each panel). NL_ECR_ and NL_TC_ denote the upper latency limits of the healthy horse group. These extremely high sensitivity and specificity values, which denote the highest possible separating power between the groups, are emphasized by the rectangular-shaped ROC plots.

**Figure 2:**
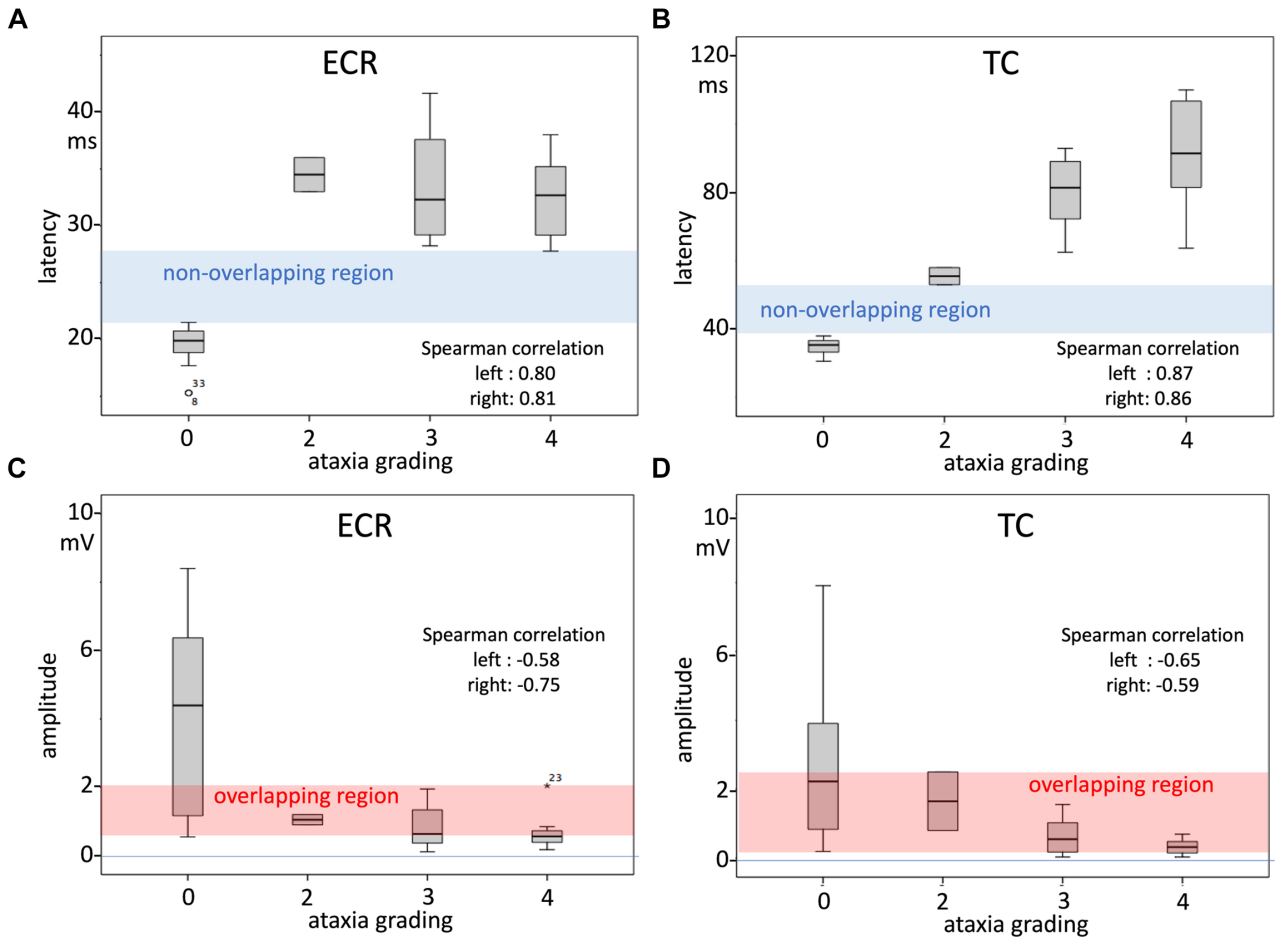
Box plots of TES-MEP latencies and amplitudes as a function of the ataxia score as an ordinal variable (**A** latencies ECR muscle; **B** latency TC muscle; **C** amplitude ECR muscle; and **D** amplitude TC muscle). The measurements represent both body sides. Spearman’s correlation factors, which are highly significant, as shown in Table 4, represent each side over the whole ataxia scale range between 0 and 4. Table 5 also shows the correlation factors for only the ataxic horse group in the range of grade 2–4 ataxia. The non-overlapping regions of the latencies of the normal and ataxic horse groups indicate that for this MEP parameter, the TES technique shows high specificity from grade 2 ataxia onwards. In contrast, the overlapping regions delineated in the MEP amplitude plots indicate that this MEP parameter shows poor specificity and sensitivity to discern between the healthy horse group and grade 2–4 ataxic group: only MEP amplitudes >2 mV or <0.55 mV for the ECR and >2.5 mV or <0.19 mV for the TC indicate per muscle respectively soundness of horses or grade 2–4 ataxic horses.

The large differences in latency times between the healthy and ataxic horse groups are clearly illustrated in Figure 1, in which panels A and B depict sensitivity/specificity curves as a function of the combined latencies of the left and right body sides, where panel A illustrates the Youden’s index YC_ECR_ for the ECR and panel B the Youden’s index YC_TC_ for the TC muscle groups. The corresponding receiver operating characteristic (ROC) graphs for TES-MEP latencies from these muscle groups in all horses are shown in Figures 1C,D. The cutoff values for latency NL_ECR_ and NL_TC_ and Youden’s cutoff YC were used to differentiate between healthy and pathologically prolonged latencies. NL and YC_ECR_ and YC_TC_ enclose non-overlapping regions of latencies between the healthy and ataxic horse groups. In addition, the box plots, depicted in Figure 2, show significantly prolonged latency times for both the ECR and TC in the ataxic horse group. It is important to notice the presence of these well-delineated non-overlapping regions between the healthy and ataxic groups for both the ECR and TC MEP latency times, indicating an explicit separation between the latencies of the ataxic group versus the healthy horses and thus underlining the sensitivity of this neurophysiological technique. Indeed, the large gap between the two distribution functions (healthy vs. ataxic) highlights the very high sensitivity of the TES technique for the detection of grades 2–4 ataxia.

The box plots in Figure 2, together with the equations of the regression lines, indicate that for the ECR and TC muscles, TES-induced MEP latency times at grade 1 ataxia most probably can be expected to be prolonged or partially overlap the normal range in a transitional zone. This expectation is confirmed by substituting grade 1 ataxia in the regression line equations in Table 5. According to Table 5, the correlation factors are highly significant over the whole range of the ataxia grading scale between 0 and 4. However, it also can be noticed in the box plot of the ECR between grades 2–4 that the latency increase is not correlated with the ataxia grading, while in contrast, for the TC, between grades 2–4, the latency increases with the ataxia grading and the correlation of the combined body sides is also significant.

**Table 5:** Regression line equations of MEP latencies and MEP amplitudes as a function of ataxia gradings (slope coefficient: A [ms/grading step]) and Spearman’s correlation coefficients (cor) and significance (sig) obtained at the left, right, and combined body sides for the ECR and TC muscles (^*^significance <5%).

MEP latency Ataxia grading 0–4							
Latency (ms)	*N*	Cor	Sig	Latency (ms)	*N*	Cor	*p*
latECR_left_ = 3.41A + 20.2	21	**0.80**	**0.000** ^*^	latTC_left_ = 14.2A + 34.4	21	**0.87**	**0.000** ^*^
latECR_right_ = 3.50A + 19.7	21	**0.81**	**0.000** ^*^	latTC_right_ = 14.0A + 34.8	20	**0.86**	**0.000** ^*^
ECR_left_ and ECR_right_	42	**0.81**	**0.000** ^*^	TC_left_ and TC_right_	41	**0.87**	**0.000** ^*^
Ataxia grading 2–4							
	*N*	Cor	Sig		*N*	Cor	*P*
ECR_left_	9	−0.28	0.46	TC_left_	9	0.55	0.13
ECR_right_	9	−0.15	0.71	TC_right_	8	0.64	0.09
ECR_left_ and ECR_right_	18	−0.16	0.59	TC_left_ and TC_right_	17	**0.61**	**0.010** ^*^

MEP amplitude Ataxia grading 0–4							
Amplitude (mV)	N	Cor	Sig	Amplitude (mV)	*N*	Cor	*P*
ampECR_left_ = −0.78A + 3.5	21	**−0.58**	**0.006** ^*^	ampTC_left_ = −0.60A + 2.7	21	**−0.65**	**0.000** ^*^
ampECR_right_= –1.1A + 4.5	21	**−0.74**	**0.000** ^*^	ampTC_right_ = −0.59A + 2.5	20	**−0.59**	**0.006** ^*^
ECR_left_ and ECR_right_	42	**−0.65**	**0.000** ^*^	TC_left_ and TC_right_	41	**−0.62**	**0.000** ^*^

Significant correlation coefficients and significance values are printed in bold.

In contrast to the high specificity of MEP latencies, the MEP amplitudes show an overlapping region between healthy and ataxic horses, with MEP amplitude values between 0.55 and 2.1 mV for the ECR and between 0.19 and 2.5 mV for the TC, as seen in Figures 2C,D, respectively. Interestingly, amplitudes above the overlapping regions apply only to the group of healthy horses. Below 0.55 mV for the ECR and below 0.19 mV for the TC, the specificity and positive predictive values (PPV) are 100%, and the sensitivities are as low as 33 and 30%, respectively. Table 6 provides an overview of cutoff MEP amplitudes and their sensitivity and positive predictive values (PPV) for detecting ataxia from the ECR and TC muscles at specificity values between 70 and 100%. Again, for TES-induced MEP amplitudes, no significant left–right differences were found, as depicted in Table 5. The descending courses of the regression lines of the MEP amplitudes are related to increasing PPVs with increasing grades of ataxia. For incrementing ataxia scores of 2, 3, and 4, the PVV increases to 0, 25, and 50% for the ECR (0.55 mV cutoff amplitude) and 0, 29, and 38% for the TC (0.19 mV cutoff), respectively. There is a low intra-individual reproducibility for the assessment of MEP amplitudes. The quotient of the mean difference between sides and the group mean m_L-R_/m indicate intra-individual reproducibilities of 10–30% fractions of high interindividual MEP amplitude variations, of which the CVs are 70–110%. This high variability agrees with the wide distribution of MEP amplitudes, as depicted in the box plots.

**Table 6:** Cutoff MEP amplitude, sensitivity, and positive predictive values (PPV) for the detection of ataxia from ECR and TC MEP amplitudes at specificity values between 70 and 100%.

Detection of ataxia from MEP amplitude specificity, cutoff amplitude, specificity, and PPV						
	ECR			TC		
Specificity	Cut off amplitude	Sensitivity	PPV	Cut off amplitude	Sensitivity	PPV
100%	0.55 mV	33%	100%	0.19 mV	29%	100%
90%	0.62 mV	56%	83%	0.44 mV	47%	73%
80%	1.0 mV	78%	78%	0.49 mV	53%	69%
70%	1.5 mV	83%	68%	1.2 mV	82%	64%

### Diagnostic imaging results versus ataxia grading

**Figure 3:**
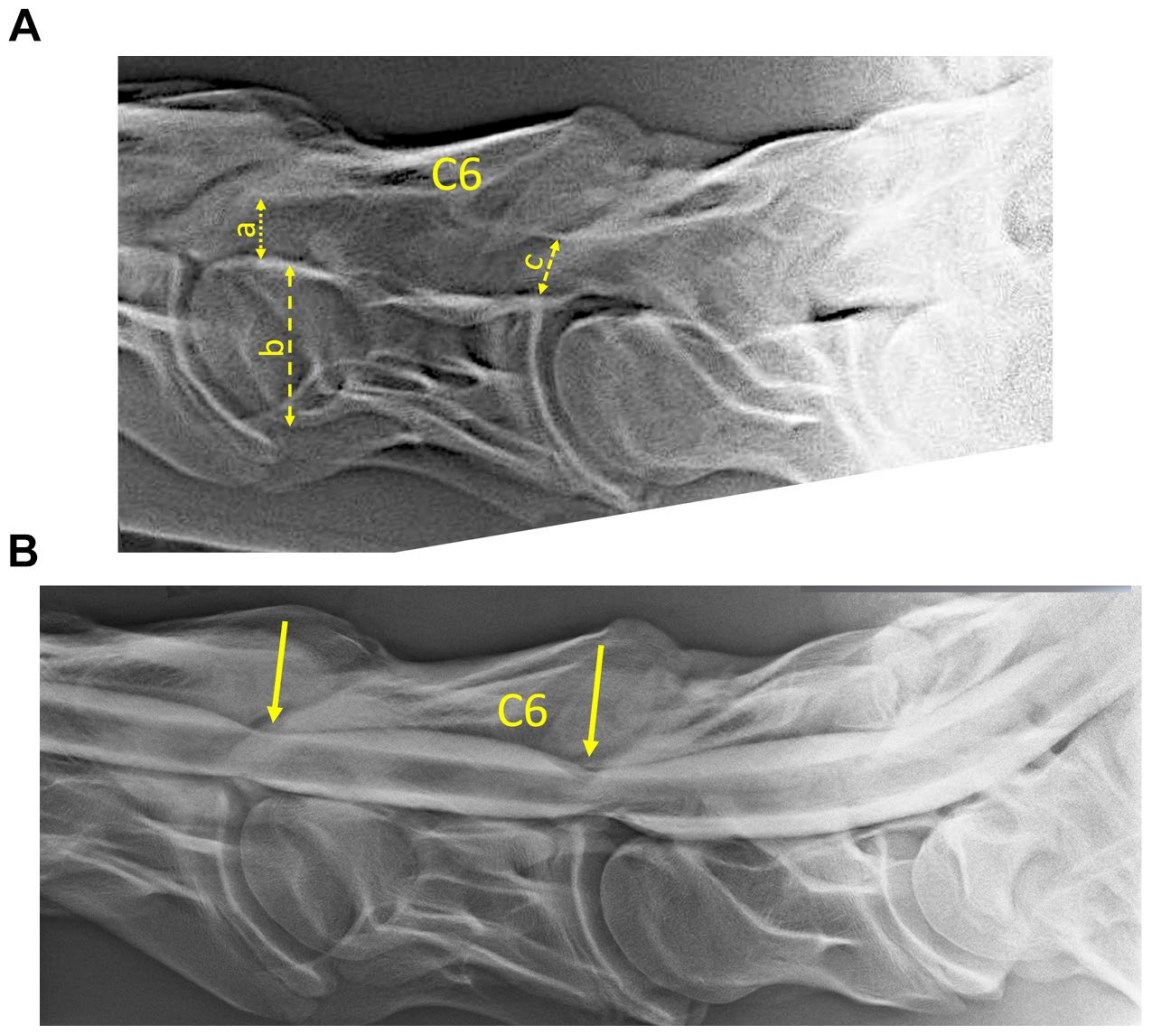
**(A)** Radiograph and **(B)** associated myelogram (neutral position) of horse H (see also Table 2), scored with grade 4 ataxia. The radiographs showed the presence of radiological signs of stenosis at levels C5/C6 and C6/C7, which were confirmed by two radiological certified observers. The connected arrows show the width of the vertebral body of C6 (b), the minimum inter-vertebral diameter (a), and the minimum intravertebral diameter (b) as defined according to Hahn et al. (2008). The inter- and intra-sagittal diameter ratios of C6, as well as the reductions of the dorsal column and the inter-vertebral dural diameters at C5/C6 and C6/C6, as indicated by the arrows in the myelogram were scored positive by two radiological observers. Pathological prolonged latencies were registered bilaterally for MEPs of the ECR and TC muscles. The segmental sites of myelum stenosis and compression are consistent with the gross pathology and observed microscopic lesions.

Table 2 provides a detailed overview of obtained medical imaging results (radiography vs. contrast myelography) per horse and across three different ataxia grading scales (grades 2, 3, and 4). Signs of stenosis were most often detected on contrast myelography (8 horses B–I) and less frequently on radiography (7 horses, B–C; E–I). Despite grade 2 ataxia and significantly increased TES-induced MEP latency times in the TR, ECR, and TC muscles, no stenosis was detected by means of medical imaging nor during gross pathology in case A. However, histopathology did show neuronal segmental damage and muscle atrophy (Table 2). Figures 3A,B show Horse H with ataxia grade 4, a cervical radiograph (Figure 3A), and the associated contrast myelogram (Figure 3B). This horse showed pathologically prolonged latency times bilaterally in the ECR and TC muscles, while the myelograms scored positive at levels C5/C6 and C6/C7.

The respective radiological and myelographic positive scores were for grade 2 (1 horse): 0 and 0, grade 3 (4 horses): 4 and 10, and grade 4 (4 horses): 10 and 12.

The interobserver reproducibility for all evaluations performed by the two observers was for the intra- and inter-segmental sagittal diameter ratio assessment 84% (*N* = 44) and 98% (*N* = 45) and for the myelographic parameters of the dorsal column and spinal diameter reduction assessment 97% (*N* = 130) and 91% (*N* = 129), respectively. For ataxia grades of 2; 3 and 4, the values are for myelography: 0; 0.69 (*N* = 16) and 0.68 (*N* = 31) and for radiography: 0; 0.25 (*N* = 4) and 0.56 (*N* = 14). The agreement between the medical imaging observers increases with the increasing degree of ataxia shown by the horses. Within each horse, myelography revealed signs of stenosis at many more sites than radiography.

When translating the medical imaging findings toward gross pathology and histopathology, important discrepancies were found. In horses B, E, and F, signs of stenosis were visible both on radiography and myelography, though no signs of stenosis could be detected during macroscopic inspection of the spinal canal. However, on all occasions in these horses, segmental histopathology revealed signs of neuronal damage and muscle atrophy.

The stenotic locations identified during medical imaging showed important discrepancies with those observed during the post-mortem observations: horse C showed a radiological stenosis at the level of C5 and myelographic at the level of C4/C5, while macroscopically, a stenosis was visible at C3/C4 and microscopically stenoses were located at C2/C3 and C3/C4. Horse E showed no macroscopic stenoses; however, it did show a histological stenosis at the skull–C1 transition.

The latencies of all muscle MEPs were significantly prolonged in all ataxic horses. In all horses, histological lesions were segmentally widespread in the spinal cord. As shown in Table 2, this is histologically recognized as the presence of Wallerian degeneration across all or nearly all segmental levels between the skull and the first thoracic spinal corpus.

The white matter abnormalities at the skull/C1 segment of horse D are observed in both ascending and descending spinal cord tracts in the ventral, dorsal, and lateral funiculi, while caudally white matter changes were present in the lateral and ventral descending tracts.

### Gross pathology and histopathology findings versus ataxia grading

Macroscopic stenotic sites were identified in five out of nine horses (C, D, G–I) (Table 2). Stenotic sites were more easily visible and identifiable with increasing grades of ataxia, in which one horse with a grade 2 ataxia showed no visible stenosis, and 2 out of 4 horses with grade 3 and 3 out of 4 horses with grade 4 ataxia showed visible stenoses. Except for one horse suffering from grade 4 ataxia showing stenoses at 5 consecutive anatomical levels, only one stenotic site could be identified in all horses. The histologically identified damaged sites corresponded with the macroscopically determined stenotic locations in all horses, except for one horse, showing a single segmental level difference between gross and microscopic pathology.

White and gray matter damage was identified in a much wider anatomical segmental range when compared to macroscopic and microscopic site identification of stenotic processes (Table 2). All pathological changes recorded in the white matter of the spinal cord were widely dispersed across all cervical segments. Gray matter damage, in turn, was more localized and delineated itself around a more specific segmental level.

In all horses, there were significantly increased latencies in all extremity muscles, and histological pathology was found in both white and gray matter. The white matter abnormalities extended over the entire cervical tract in all degrees of ataxia, except for two horses in which the cervical spinal tract damage still extended over 4 and 6 multiple segments, respectively. The gray matter was usually affected over a smaller area ranging from 1 to 7 segments. In some cases, stenosis was seen on medical imaging that could not be confirmed either macroscopically or microscopically. In 2 horses (horses B and F), no stenotic site could be identified, gross macroscopically, though both radiographic and myelographic recordings were scored as positive for the presence of stenosis. In one of these horses, the stenosis, visible on medical imaging, existed in conjunction with a narrow spinal canal, severe osteoarthrosis of an articular process joint with bony proliferation into the spinal canal, and shape variations of the articular process joints. The segmental locations of histopathological changes consistent with stenoses at static compression were most consistent with the myelography results and usually covered the region ranging from C5 to T1 and at least C6/C7 and concerned horses D, F, H, and I. In a smaller number of cases, these were also detected on radiography (horses H and I) or during gross pathology (horses D, H, and I). In horse G, a dynamic stenosis was found at the level of C3/C4 and C4/C5, with the neck in a flexed position (Table 2). *Post mortem*, spinal stenosis was most often detected on histopathology (Table 2: horses B–E, G–I; 7 horses) and to a lesser extent during gross pathology (visual inspection) (horses C, D, G–I; 5 horses).

**Figure 4:**
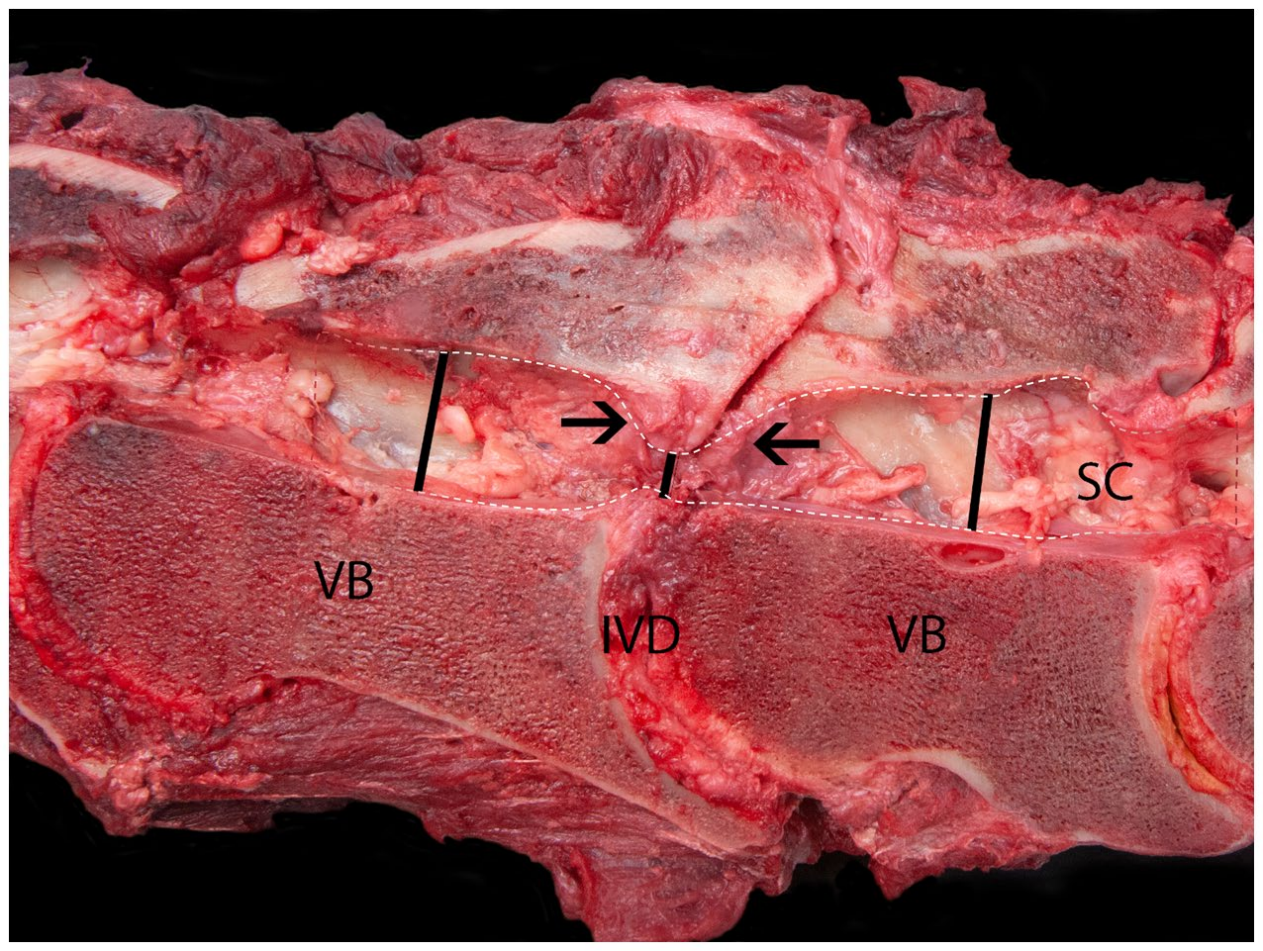
Sagittal cut of the vertebral column at C6/C7 of a 12-years old Dutch warmblood horse with static cervical vertebral compressive myelopathy. The spinal canal (SC) at the level of the C6/C7 articular process joints is severely narrowed (short line) due to the proliferation of bone (arrows) originating from both degenerated articular process joint facets. VB, vertebral body; IVD, inter-vertebral disk. The lines show the diameter of the spinal canal cranial, caudal, and at the site of the bony proliferation.

Figure 4 provides an example of multiple visually identifiable spinal canal stenoses at the level of C5/C6 and C6/C7 on a sagittal cut of the vertebral column of a 20-month old Dutch warmblood horse (Horse H). The bony boundary of this stenosis is delineated by means of a dashed line. This is a static vertebral compressive myelopathy. The spinal canal (SC) at the level of the C6/C7 articular process joints is severely narrowed (short line) due to the proliferation of bone (arrows) originating from degenerated articular process joint facets at both locations. The two lines show the larger diameters, rostral and caudal, from the location of the bony proliferation.

**Figure 5:**
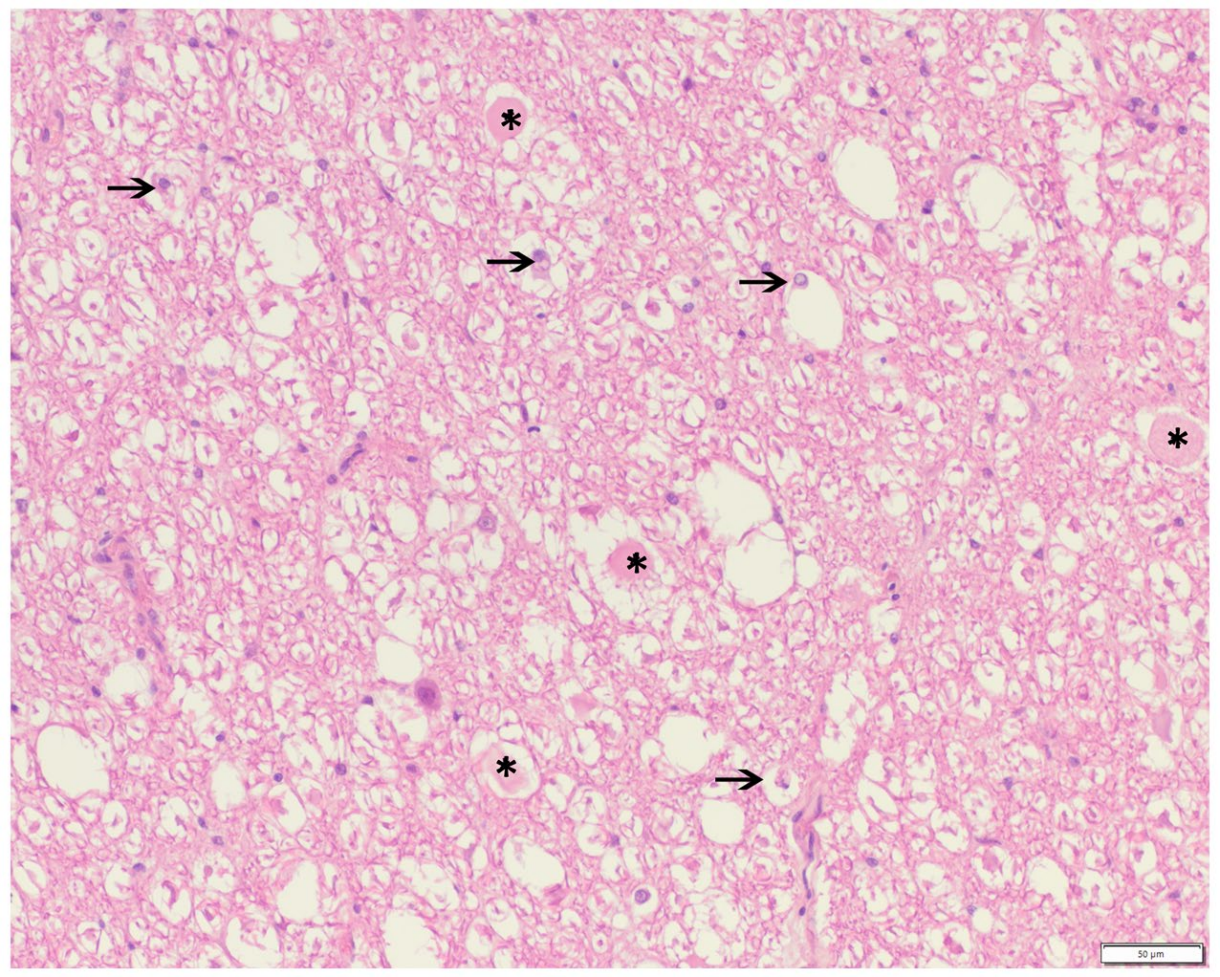
Haematoxylin and eosine stain of a formalin-fixed spinal cord cross-section at the level of the inter-vertebral articulation between C6 and C7 of a 2-year old Groninger horse (horse G) with cervical vertebral compressive myelopathy showing the presence of multifocal dilated myelin sheaths with swollen axons (*, spheroids) or macrophages (arrow, digestion chambers), consistent with Wallerian degeneration.

In all examined horses, histopathology revealed the presence of pathological changes in both the white and gray matter and at the level of the spinal nerves (Table 2). The changes in the white matter were most widely distributed. In eight out of nine horses, a site of compression could be established histologically, and this site corresponded in 5 out of 9 horses with the grossly visible compression site. In one horse (D), the compression sites identified during either gross or histological examination were one segment apart. In one horse (F), in which no macroscopic compression site could be identified, the histological changes were compatible with equine degenerative myeloencephalopathy with, besides the presence of severe damage at the level of both the white and gray matter, also severe brain stem damage. All horses showed histological changes compatible with denervation atrophy in varying degrees across the studied muscles (Table 2). In Figure 5 an example of severe Wallerian degeneration is visible, found in horse F (Table 2), showing histological changes in the spinal cord at the level of the inter-vertebral articulation between C6 and C7. Visible are multifocal dilated myelin sheaths with swollen axons and macrophages, compatible with Wallerian degeneration.

In 5 horses (horses B–E, G, I), the stenoses were noticed at the level of only 1 or 2 segments. In horse (H), microscopic stenoses were present at three levels. In 4 horses, the location of either grossly or histopathologically identified stenoses was largely consistent with the medical imaging findings (horses D, G–I) (Table 2).

No stenoses were observed in case A, neither with diagnostic imaging nor during pathomorphological evaluation.

White matter degeneration was observed in all cases and was, with the exception of case B, widely spread across almost all segments. Only case B showed moderate degeneration across a smaller delineated area between skull/C1 and C2/C3.

Gray matter degeneration was noticed in all cases. In the case of F, there was, besides severe gray matter degeneration in the thoracic segment, also severe degeneration visible in the brain stem. In three cases, the gray matter degeneration was restricted to 1 segment (B, C, and G), in 1 case to 2 segments (A), distributed over more than 2 segmental levels in the remaining 4 cases (D, E, H, and I), and in 1 case (F) only in the brain stem and a thoracic segment.

When enough time has elapsed, cascaded effects via motoneurons and segmental nerves may become histologically apparent, such as muscle atrophy. Changes compatible with nerve compression and muscle atrophy were observed in all cases and were, except for case B, usually widely spread across the entire cervical segmental range. Case B showed at the segmental junction of C7/T1 only for the right segmental nerve a score of 2 for degeneration.

**Figure 6:**
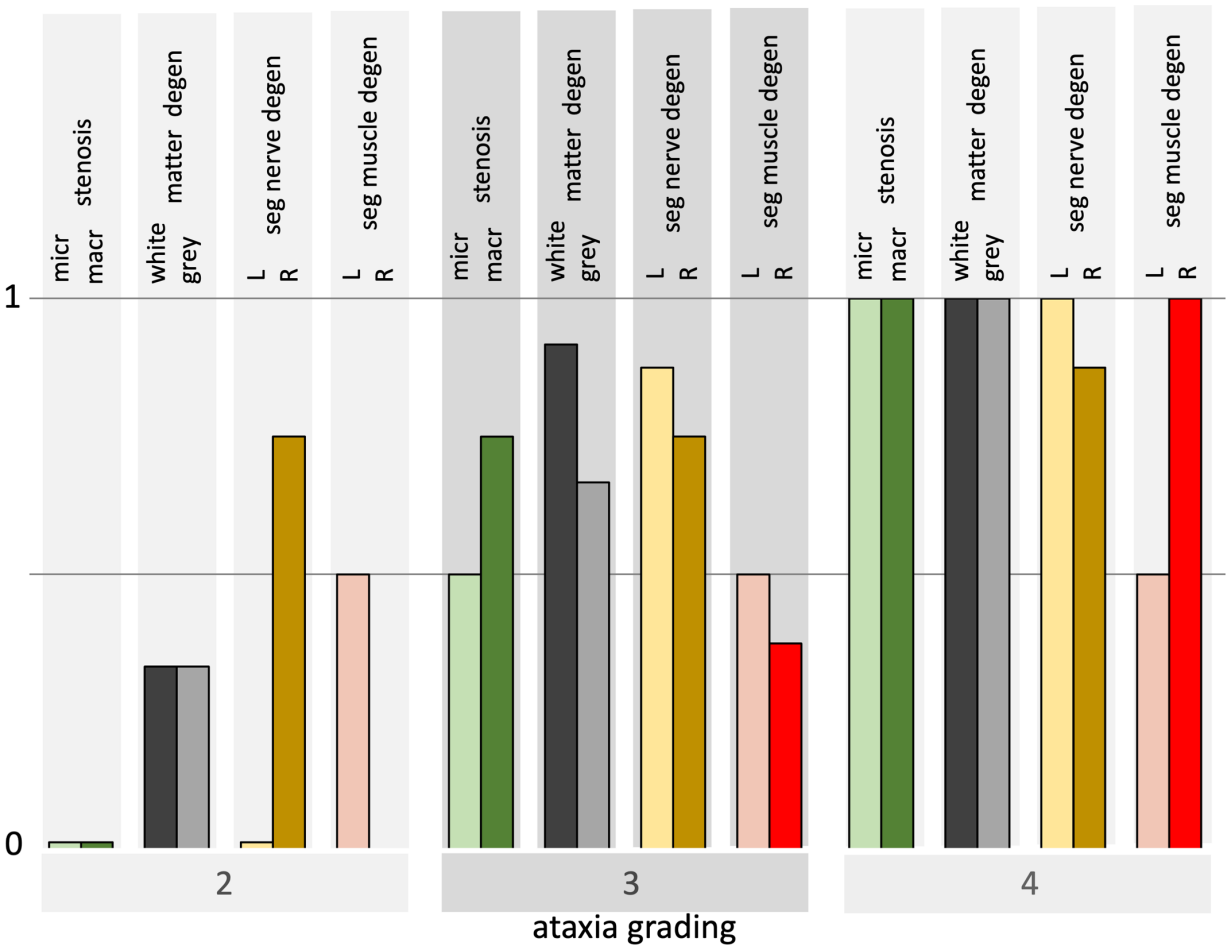
Bar graph overview of histological findings for horses from grades 2–4 ataxia. Each bar at grades 3 and 4 represents the average over 4 horses of the necropsy parameters which are normalized to their overall maximum for both sides. The histological parameter outcome per ataxia group is illustrated from left to right: the presence of macroscopic and microscopic stenosis, white and gray matter degeneration, left and right segmental nerve degeneration, and left and right muscle atrophy.

Figure 6 summarizes the histology parameters depicted in Table 2 and illustrates the dependence of post-mortem parameters on the degree of ataxia. The annotated scores for degeneration of the white matter, gray matter, segmental nerves, and segmental muscles are averaged over the 4 horses in the ataxia grades 3 and 4 subgroups after normalization to the overall maximum score. Spinal cord compression was absent at ataxia grade 2, showed intermediate values around 0.5 at grade 3, and reached a normalized score of 1 at grade 4 ataxia. The white and gray matter degeneration lowest score was 0.3 in grade 2, 0.9 and 0.7 in grade 3, and reached a normalized score of 1 in grade 4 ataxia. The scores of the segmental nerve degeneration and muscle atrophy illustrate the widespread dispersed presence of histological changes at all ataxia grades. The scores show increasing values with increasing grades of ataxia.

In most horses, bilateral segmental nerve and denervation damage was observed with symmetrical presence in 1 or more segments. This concerns the segmental nerves in horses B–E (ataxia grade 3), F–I (grade 4), and the segmental muscles in horses C (grade 3) and F–I (grade 4). It should be noted that this segmental symmetry occurs mainly in horses with the highest ataxia score of 4. In general, the degeneration in the segmental nerves was more widely spread over cervical levels than the segmental muscle atrophy.

## Discussion

The current study is the first to combine a multitude of ancillary diagnostic techniques to underpin the specificity and sensitivity of a transcranial stimulation technique, in this case, TES, to assess neuronal functional integrity in horses. Study results clearly show a high diagnostic reliability of the TES technique, especially with respect to the assessment of TES-induced MEP latency times in the ECR and TC. TES-induced MEP amplitudes are less reliable. No left–right body side differences were detected in the recorded TES latencies and amplitudes, not in the healthy horse group, nor the ataxic horse group.

### Sensitivity and specificity of latency times

Study results reveal a high sensitivity and specificity for the TES technique to assess spinal cord functional integrity in ataxic horses. The sensitivity and specificity of the recorded latency times in the ataxic horses yield, according to the ROC plots in Figures 1C,D, the highest possible values of 100%, whereas the distribution functions of the healthy and ataxia groups are clearly separated by a non-overlapping region as shown in Figures 2A,B for the ECR and TC. The Youden’s cutoff point for the ECR YC_ECR_ is 24.6 ms for the detection of pathological latency times with a sensitivity of 100% and specificity of 100%. This is well above the 95% upper bound of the distribution function of the healthy group, NL_ECR_ = 21.5 ms and maximum 21.8 ms. Similarly, Youden’s cutoff point for the TC: YC_TC_ = 45.5 ms with a sensitivity of 100% and specificity of 100%, whereas the upper bound distribution function of the healthy group is NL_TC_ = 37.2 (95%), with a maximum of 37.4 ms. The positive biases of Youden’s cutoff points (YC) compared to the NL values are, as expected, related to the presence of the non-overlapping regions of latencies of the healthy and ataxia groups and is the reason for using the NL values as cutoff in the current study.

The convincing discriminative ROC curves in Figure 1 were derived according to Youden’s approach to determining the latency cutoff values. The non-overlapping regions between healthy and ataxic horse latencies illustrate the high sensitivity and selectivity (100%) of the TES technique to discern between healthy and ataxic horses, as of a grade 2 ataxia onwards. A similar approach was described in a study investigating another transcranial stimulation technique: transcranial magnetic stimulation (TMS), first applied on horses by Mayhew and Washbourne (1996) and Rijckaert et al. (2019). These researchers described latency time cutoff values for transcranial magnetic stimulation (TMS)-induced muscle MEPs recorded in the ECR and TC of a group of 19 ataxic horses, including one horse with grade 1 and none with grade 2 ataxia (Rijckaert et al., 2019). That study reports Youden’s cutoff values to detect spinal cord dysfunction of 22 ms with a sensitivity (95% confidence interval) of 88% [73–100%] and a specificity of 100% [100–100%] for the ECR and 40 ms with a sensitivity (95% confidence interval) of 94% [83–100%] and a specificity of 100% [100–100%] for the TC. The biases of Youden’s cutoff values are 1–4 ms lower than in the current study.

In the current study, we cannot yet make solid statements about grade 1 ataxia due to the lack of involvement of that ataxia grade.

The hypothesis that latencies are prolonged in ataxic horses is thus confirmed by the study results, though it should be mentioned that the current study involves a limited number of horses.

### Positive predictive value (PPV) of TES-MEP latencies and medical imaging versus ataxia grading

**Figure 7:**
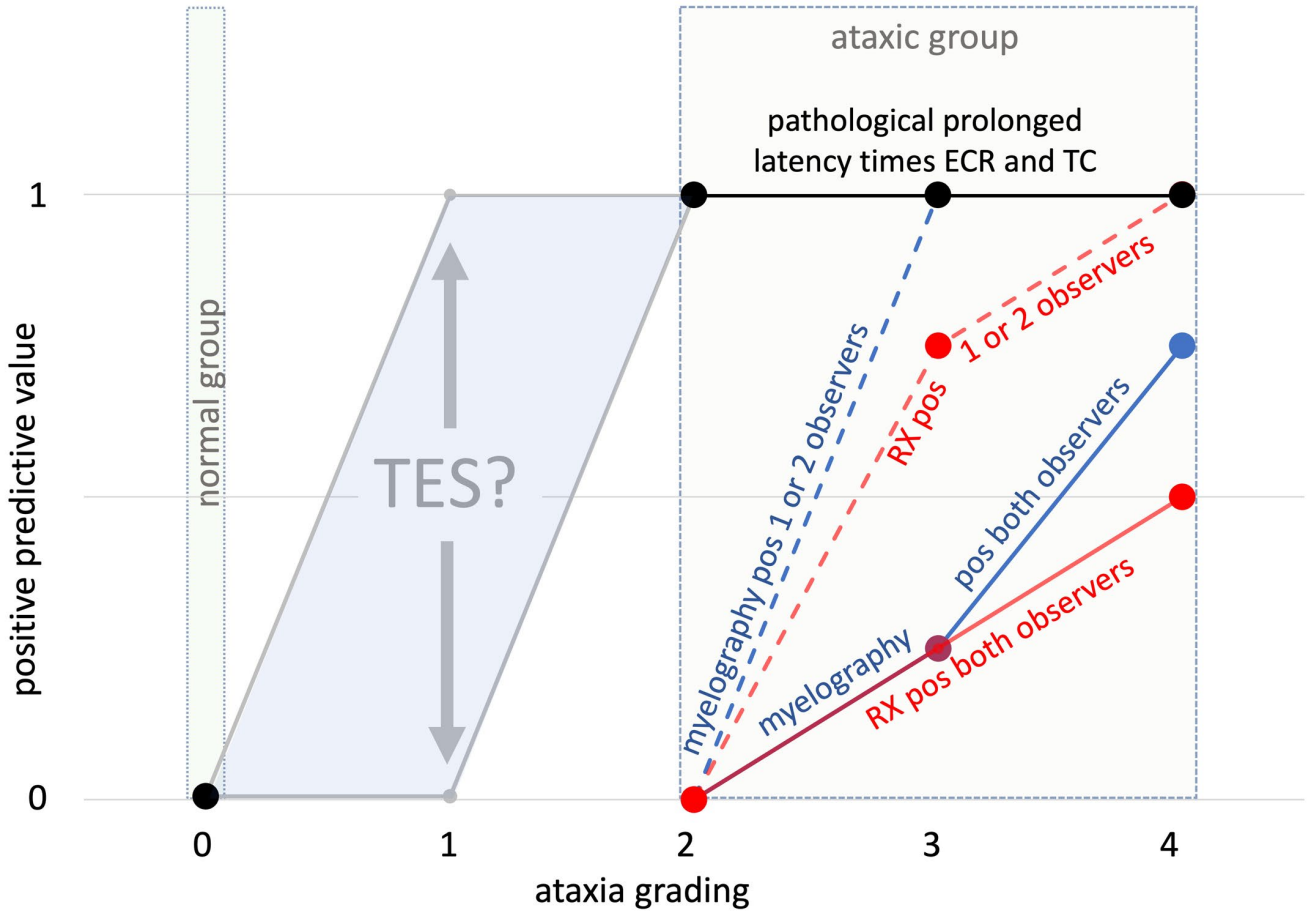
Overview of calculated positive predictive values (*y*-axis) for TES-MEP latencies per ataxia grade (*X*-axis) and medical imaging results (RX vs. myelography). The dotted lines represent horses in which either myelography (blue dotted line) or radiography (red dotted line) was deemed positive for the presence of spinal stenosis by at least one of two certified observers. The solid lines represent horses in which either myelography (blue solid line) or radiography (red solid line) was deemed positive for the presence of spinal stenosis by both certified observers. Because of the lack of horses with grade 1 ataxia involved in the current study, the gray colored PPV zone delineates the range of “predicted” PPVs for horses with grade 1 ataxia. The question sign indicates that a measured PPV at grade 1 is not available in this study.

When the PPVs of TES-MEP latencies and medical imaging are compared as a function of the different ataxia grades ranging from 0 to 4, it becomes clear that medical imaging techniques need to be viewed as ancillary diagnostic techniques that basically are not suitable to assess neuronal functional integrity. Figure 7 shows that for all ataxic horses, PPVs are 100% for the ataxic group and 0% for the healthy group for the recorded TES-induced latency times. Due to the lack of horses with grade 1 ataxia included in the current study, a preliminary PPV zone for this grade was created, covering every possible PPV between 0 and 100%. The inclusion of horses suffering from grade 1 ataxia in future studies will allow for further fine-tuning of latency time cutoff values. More research is needed in that respect. However, it can be expected that horses suffering from grade 1 ataxia most often have a better prognosis and thus will not be available for the performance of post-mortem examination.

The study results also clearly show lower PPVs for medical imaging techniques when compared to TES-MEP latencies. As indicated by the segmental profiles of RX and myelography, PPVs are for myelography for ataxia grades 3 and 4 higher when compared to plain radiography and become 100% for grade 4 ataxia cases when scored positive by minimally 1 observer.

### Correlation between MEP latency and ataxia

The MEP latency increases recorded at the level of the ECR show a low correlation with ataxia gradings, which entails that in this muscle, there is no clear-cut correlation between the degree of latency time increase and the grade of ataxia shown by the horse. In the TC, however, there is a clear and high correlation, which means that the degree of latency time increases recorded at the level of the TC follows an increasing trend with increasing grades of ataxia. A possible explanation for this finding is the longer conduction route represented by the TC, which contributes to the expression of more pronounced increased latency times with increasing degrees of ataxia. Table 5 shows, over the full ataxia range (0–4), highly significant large correlation factors.

The correlation of the ECR latencies is about zero and is independent of the ataxia grading, which is also visible in the box plot of Figure 2A. Conversely, for the TC, the latencies increase with increasing ataxia grading (Table 5 and Figure 2B).

### Correlation between MEP amplitude and ataxia

The recorded amplitudes depicted in Figures 2C,D and Table 5 show decreasing values with increasing ataxia grades for the ECR and TC muscle groups with highly significant Spearman’s correlations between −0.58 and − 0.65 and between −0.65 and − 0.59 for the ECR and TC, respectively. However, there is an important overlapping of amplitude values between healthy and ataxic horses, rendering the TES-induced MEP amplitudes markedly less reliable than latencies to assess neuronal functional integrity in horses.

### Comparison of MEP latencies and amplitudes between sides

When comparing body sides, no differences were detected in recorded latency times. The latencies of the regression line equations in Table 5 and their slope coefficients, mean values of the latencies, and amplitudes are statistically equal to each other. The latency time value for the right side of horse C is missing in Table 4 because it could not be reliably determined due to a dispersed small TC MEP response at that side. The symmetry may possibly be ascribed to the mostly bilateral wired extrapyramidal motor tracts in horses missing the dominating unilateral corticospinal connections, which are present in primates and humans.

When asymmetries in MEP latencies and amplitudes are encountered, these most likely result caudally from an asymmetric stenotic process as described in a case report of a horse suffering from a right-sided C4C6 joint disease associated with the recording of right-sided prolonged latencies of ECR and TC-TMS-induced MEPs (Bailey et al., 2022).

### Sensitivity and specificity of MEP amplitudes

In contrast to the high sensitivity and specificities of latencies, TES-induced MEP amplitudes are less sensitive to discern between healthy and ataxic horses due to the marked overlap of TES-induced MEP amplitudes between healthy and ataxic horses, as shown in Figures 2C,D. Table 6 depicts the ECR and TC at a specificity of 100% (amplitude cutoff values: 0.55 mV and 0.19 mV), PPVs of 100%, and sensitivities of approximately 30%, while at a specificity of 70% (cutoff values: 1.5 and 1.2 mV), PPVs decrease to ~66%, and sensitivities increase to 82%. Figures 2C,D indicate that the discriminative power of MEP amplitudes, similar to the radiological assessment, only becomes evident at the high ataxia grades 3 and 4.

### Sensitivity of TES-MEP latencies compared with radiological assessment

All involved ataxic horses showed increased latency times in both the ECR and TC muscles. However, not all of them showed signs of stenosis on medical imaging. A higher prevalence of stenosis was seen in myelography when compared to radiography, and there was a higher accordance between observers for myelographic assessment (68%) when compared to radiography (56%). The latter could be explained by the more straightforward identifiable stenosis parameters on myelographic imaging than radiographic recordings, which show a lot of superposition of anatomical structures. This greater inter-observer accordance for myelography when compared to radiography has also been reported by other research groups.

The sensitivity and specificity of intra- and inter-sagittal diameters for detecting myelum compression are, according to Hughes et al. (2014) lower than dorsal column and dural diameter ratios (Van Biervliet et al., 2004). These qualitative differences also emerge in this study, as can be seen in Figure 7. The sensitivities to detect stenoses found in the current study are somewhat higher than reported in the literature. Van Biervliet et al. (2004) reported a sensitivity of 53% (34–72%; 95% confidence interval) for myelograms. Hughes et al. (2014) reported radiological inter-vertebral sagittal ratio assessment (SRV) at a cutoff value of 48.5%, a rather poor sensitivity of the method of 20% (median), and median specificity of 100% when 4 observers were involved (Hughes et al., 2014). The mean sensitivity increased to 69% when changing cutoff values to 50% for the C3–C6 segment and 52% for C7 at the cost of a specificity decrease from 100 to 61%. Levine et al. (2007) reported for intravertebral sagittal ratio assessment a sensitivity of 47% and a specificity of 78% for detecting lesions (Levine et al., 2007). Figure 7 shows PPVs of 100% for MEP latencies at ataxia grades 2 and above. These PPVs are significantly higher than the radiological PPVs, whose values remain 0 until ataxia grade 3. This underlines the important value of the TES technique for clinicians to assess the neuronal functional integrity of their patients. Interestingly, the medical imaging inter-observer reproducibility is higher at high levels of ataxia (grades 3 and 4) when compared to the overall inter-observer reproducibility of 84–98% for radiological assessment and 91–98% for myelographic assessment in the current study. This is grossly consistent with published data (Hughes et al., 2014). The radiograph and associated myelogram of horse H (ataxia grade 4), depicted in Figures 3A,B illustrate positive radiological scores assigned to two adjacent cervical segments. In this horse, prolonged latency times were recorded in the muscle groups in the extremities. The sensitivity of radiological assessment to detect spinal cord pathology is low. In horse A, prolonged latency times were recorded in all involved muscles, though no spinal cord stenoses could be detected during medical imaging nor during gross pathology. The sensitivity and specificity of CT-myelography and MRI are higher when compared to RX and myelography (Lindgren et al., 2020; Rovel et al., 2021). However, CT scans of the cervical spine are generally not able to be performed with the neck in a flexed or extended position, hampering the identification of dynamic stenotic processes (Garrett, 2022).

The results of the current study also clearly show a high sensitivity of the TES technique at low ataxia grades such as grades 2 and 3. To have a clearer view of the diagnostic value of the technique for grade 1 ataxia, more horses with low-grade ataxia need to be tested; however, clearly, in most of these cases, horse owners will not opt for euthanasia, precluding the opportunity to execute post-mortem gross pathology and histopathology as a control. However, when given the predicted prolonged mean latencies at such low ataxia grades, it can be expected that TES also will proof to be a solid diagnostic technique to evaluate neuronal functional integrity at low grades of ataxia.

### Gross pathological and histological findings

Macroscopic stenotic sites were identified in five out of nine horses. It is a challenge for clinicians to estimate the clinical importance of stenotic sites identified either in radiography or myelography. Levine et al. (2007) reported in their study that 2 out of 7 horses showed signs of subluxation and compression due to the presence of articular process osteophytes on medical imaging, though no lesions were detected during necropsy at these sites (Levine et al., 2007). All horses showing macro/micro signs of stenosis also showed histological signs of neuronal degeneration and muscle atrophy (Table 2). The latter was present in all horses involved in the current study. All these horses showed significantly prolonged TES-induced MEP latency times, emphasizing the sensitivity of the TES technique to evaluate the functional integrity of the spinal cord.

As previously mentioned, white and gray matter damage was recorded in a much wider anatomical segmental range when compared to macroscopic and microscopic site identification of stenotic processes (Table 2). All pathological changes recorded in the white matter of the spinal cord were widely dispersed across all cervical segments. Gray matter damage, in turn, was more localized and delineated itself around a more specific segmental level. The more widespread manifestation of white matter damage compared to gray matter damage most probably can be explained by the fact that white matter abnormalities concern both the motor and sensory tracts that run along a stenotic focus in, respectively, a caudal and rostral direction across several segments. Therefore, white matter damage assessment does not allow for the exact determination of functional stenosis location. A more accurate determination of the focal compression site becomes feasible when the anatomical distribution of the ascending and descending tracts is taken into account. The scores globally represent the myelum, not specific spinal motor tracts in the ventral and lateral funiculi, but also dorsal sensory tracts, including proprioceptive axons. In cases suffering from lateral compression, for example, the more superficial anatomical location of the lateral proprioceptive and spinocerebellar tracts have been reported to be most vulnerable (De Lahunta, 1983; Andrews and Adair III, 1999). It is plausible that this also applies to superficial thick motor axons located in the ventral, lateral, and dorsal funiculi.

Most probably, the long segmental trajectory of white matter degeneration is also responsible for the manifestation of significantly prolonged TES-induced MEP latency times recorded in both the front and hind limbs of all involved ataxic horses. Myelin degeneration or loss is reported to be most pronounced in the ventral and lateral funiculi (Yovich et al., 1991). Myelin degeneration follows a caudal descending direction from the lesion site, while the degeneration of axons in sensory tracts follows an ascending direction (Summers et al., 1995).

The radiologically and grossly detected spinal cord stenoses are usually limited to fewer segments than the segmental extension of the white matter damage. In the horses where lesions consistent with stenosis of the spinal cord have been observed, sufficient time of at least a couple of days has apparently allowed Wallerian degeneration to spread over a larger cervical range. The instantaneous changes observed during intra-operative neuromonitoring usually encompass MEP amplitude reductions or even disappearance, while latency time increases of 10% are mostly used as alarm criteria (MacDonald et al., 2013). These changes are usually reversible when the cause can be taken away and are most likely unrelated to Wallerian degeneration since the latter will only occur a few days after the onset of stenosis. When the changes would linger for a couple of days, Wallerian degeneration may become visible as well. An accurate determination of a stenotic location with functional impact may sometimes be quite a challenge as the same lesions can also be present without actual functional impact, or they can only manifest themselves dynamically (dynamic cervical vertebral stenosis myelopathy: CVSM), or accidental destruction of the cause of compression during necropsy could have occurred (e.g., perforation of a synovial cyst).

The degeneration in the spinal cord can become visible in the nerves of mice from 3 days onwards. Denervation atrophy of muscles can evolve very quickly. However, it is unknown how fast this happens in the spinal cord upon the impact of a lesion. Due to the failure of activation of motoneurons at sites with sufficiently severe degeneration in the myelum, subsequent secondary histologically degenerative changes in nerve and muscle may then arise when motoneurons remain persistently inactive. Given sufficient time, cascaded effects acting via motoneurons may also account for the degenerative histological observations in the segmental nerves and muscles further away from the stenotic site. A segmental nerve can, of course, also be primarily affected by the stenosis. Wallerian degeneration of the motor axons proceeds caudally (Nout and Reed, 2003).

In the current study, bilateral segmental nerve and denervation damage showed a symmetrical pattern in most cases and was present in one or more segmental nerves in horses with ataxia grades 3 and 4 and the segmental muscles in one of 4 horses with grade 3 and all horses with grade 4 ataxia. The degeneration in the segmental nerves is usually more noticeable and spreads out across more segmental levels when compared to the manifestation of segmental muscle atrophy.

As shown in Figure 6 and Table 2, it is expected that an increasing number of segmental nerves and muscles will show histological abnormalities, with an increasing degree of ataxia. However, it remains a question why muscular dystrophy of the ECR and TC muscles is often less severe in the more ataxic cases. At grade 2, all 6 muscles showed mild-to-moderate signs of atrophy; at grade 3, this was the case in 1/3rd to half of the muscles; while at grade 4, severe changes were observed in only 0% to 1/6th of the muscles. An explanation for this paradox cannot yet be given.

Clinicians should be aware of the fact that neuronal and muscular damage may be extensively present without radiographic or myelographic indications of the presence of stenotic processes. Additionally, in all involved cases where stenotic processes were identified, the histological neuronal and muscular damage extended over a much larger anatomical segmental area than the stenotic site itself.

### Expanding the diagnostic range in the cervical region by including the trapezius muscle

The trapezius muscle showed a mixed pattern of normal and prolonged TES-induced MEP latency times, with normal latencies in 6 out of 9 ataxic horses. This can probably be explained by the fact that most stenoses in the examined horses occurred caudal from the cervical C2–C4 segmental root levels of the trapezius.

The inclusion of MEPs induced in muscles innervated by more cranial cervical segments makes it possible to delineate a lesion there or more cranially. Prolonged latencies of the trapezius MEPs suggest a cause of high cervical compression up to the level of the foramen magnum, an intracranial space-occupying process, or an infection or a disorder of the nervous system for which the imaging techniques used are inadequate. To avoid interpretation errors due to segmental overlap with the C2–C4 levels of the trapezius with a well-known common dynamic compression site at the C3–C4 segmental level (Powers et al., 1986; Wagner et al., 1987), cervical higher cranially innervated muscles that are innervated by more cranial cervical segmental levels could be involved in future studies. The cranial part of the splenius muscle, for example, could serve as a solid target for that purpose.

Horse I is a typical example of grade 4 ataxia with a radiological and histological clearcut compression at C5/C6 level with Wallerian degeneration in ascending and descending spinal tracts, segmental denervation, and muscular atrophy where only MEP latencies in the TC and ECR and not in the TR were significantly prolonged, but normal in the trapezius muscles (Horse I in Table 2). This level showed a severely narrowed bony spinal canal, as depicted in Figure 4, and corresponded to unanimous pathological scores from two radiological observers of the inter- and intra-vertebral diameter ratios, sagittal myelum diameter, and dorsal column reductions.

Of the three remaining horses with marked delayed trapezius MEP latencies and reduced MEP amplitudes, case E with moderate ataxia (Table 2) represents a case in which there is no match between medical imaging and histopathology findings. In this horse, widespread myelum compressions were seen on medical imaging between C1 and C6–C7 levels, while widespread moderate white matter axonal degeneration and mild gray matter degeneration between skull and C2 and between C3 and C5 are found at the transition from the skull to C1. Specifically, these high cervical levels are consistent with the delayed trapezius latencies.

Case B, with moderate ataxia, high cervical dynamic stenoses, and a myographic stenosis at C6/C7, has moderate white matter axonal degeneration from the skull and 3 contiguous segmental junctions.

In case A, all 3 muscle groups showed prolonged latencies without evidence of spinal cord compression along the entire cervical trajectory between the skull and the first thoracic vertebral body. In this horse, histopathology revealed the presence of neural damage consisting of moderate Wallerian degeneration at the level of both ascending and descending spinal tracts across the whole cervical spinal cord, without identifiable sites of compression. Furthermore, there were signs of muscle atrophy at several segmental levels, as well as denervation atrophy in the assessed muscles. More likely, this could pertain to a generalized disorder of the nervous system, such as neuroaxial dystrophy or EDM. No histological abnormalities of the white matter were observed in the brain stem of this horse.

## Conclusion

TES-induced MEP latency times are a reliable parameter of the TES technique to evaluate neuronal functional integrity. This certainly applies to horses with an ataxia grade equal to or above 2. Additional data are needed to determine the diagnostic value of the TES technique for grade 1 ataxia horses, but everything indicates that the technique is also a reliable tool for these cases. The study has shown that prolonged TES-induced MEP latency times are also recorded in ataxic horses, in which medical imaging techniques do not reveal stenotic processes, while in all these cases, extensive histological neuronal damage was seen, as well as segmental muscle atrophy. Therefore, TES is a very sensitive *in vivo* technique to evaluate neuronal functional integrity.

Study limitations

- For a proper judgment of the sensitivity and specificity of the TES technique to assess neuronal functional integrity in cases suffering from low-grade ataxia, more horses with grade 1 ataxia need to be involved in future studies.
- No radiological and post-mortem histological data were available for the healthy group. In the current study, obtained latency times were not studied/corrected for possible dependency on withers height.

Study highlights

- Histological white matter abnormalities are always present in case of significantly prolonged MEP latencies of all leg muscle MEPs.
- The given limited data set of this study shows a high sensitivity and specificity of transcranial MEP latencies of limb muscles in grades 2–4 ataxic horses, whereas positive predictive values are greater than the medical imaging outcome.
- MEP latency times of all limbs reveal a strong separation between healthy and ataxic horses.
- The addition of latency measurements of upper cervical innervated muscles offers the possibility to trace functional lesions of the spinal cord over the cervical trajectory.
- Muscle MEP amplitudes from TES have a modest diagnostic significance for only higher grades of ataxia.

## Data availability statement

The raw data supporting the conclusions of this article will be made available by the authors, without undue reservation.

## Ethics statement

Ethical approval was not required for the studies involving animals in accordance with the local legislation and institutional requirements because horses were submitted for clinical diagnostic evaluation. Written informed consent was not obtained from the owners for the participation of their animals in this study because the submitted horses were studied after verbal approval from the horse owners who also gave their permission for necropsy.

## Author contributions

SJ: Conceptualization, Data curation, Investigation, Methodology, Validation, Visualization, Writing – original draft, Writing – review & editing, Formal analysis. HJ: Conceptualization, Data curation, Formal analysis, Investigation, Methodology, Software, Supervision, Validation, Visualization, Writing – original draft, Writing – review & editing. WB: Data curation, Methodology, Validation, Visualization, Writing – review & editing. IC: Data curation, Formal analysis, Methodology, Visualization, Writing – review & editing. KV: Data curation, Validation, Writing – review & editing, Methodology. ER: Data curation, Validation, Methodology, Writing – review & editing. SR: Conceptualization, Formal analysis, Investigation, Methodology, Validation, Visualization, Writing – review & editing. CB: Data curation, Investigation, Methodology, Validation, Writing – review & editing. HB: Conceptualization, Data curation, Formal analysis, Methodology, Validation, Visualization, Writing – review & editing. CD: Conceptualization, Formal analysis, Funding acquisition, Investigation, Methodology, Project administration, Supervision, Validation, Visualization, Writing – original draft, Writing – review & editing.

## References

[ref1] Andrews F. M. Adair H. S. III (1999). “Anatomy and physiology of the nervous system” in Equine Surgery. eds. Auer J. A. Stick J. A. (Philadelphia: W.B. Saunders Co.), 405–412.

[ref2] Bailey J. Bowen I. M. Anghileri B. Baiker K. Henson F. M. D. (2022). Unilateral degenerative joint disease of a cervical articular process joint between the fourth and fifth cervical vertebrae causing asymmetrical ataxia in a young horse. Equine Vet. Educ. 34, E268–E273. doi: 10.1111/eve.13579

[ref3] Bedenice D. Johnson A. L. (2022). Neurologic conditions in the sport horse. Anim. Front. Rev. Mag. Anim. Agric. 12, 37–44. doi: 10.1093/af/vfac036, PMID: 35711509 PMC9197298

[ref4] De Lahunta A. (1983). Veterinary neuroanatomy and clinical neurology. 2nd. Philadelphia: W.B. Saunders Co.

[ref5] Duncan I. D. Schneider R. K. Hammang J. P. (1987). Subclinical entrapment neuropathy of the equine suprascapular nerve. Acta Neuropathol. 74, 53–61. doi: 10.1007/BF00688338, PMID: 3661120

[ref6] Einarson L. (1953). Deposits of fluorescent acid-fast products in the nervous system and skeletal muscles of rats with chronic vitamin-E deficiency. J. Neurol. Neurosurg. Psychiatry 16, 98–109. doi: 10.1136/jnnp.16.2.98, PMID: 13053230 PMC503120

[ref7] Estell K. Spriet M. Phillips K. L. Aleman M. Finno C. J. (2018). Current dorsal myelographic column and dural diameter reduction rules do not apply at the cervicothoracic junction in horses. Vet. Radiol. Ultrasound 59, 662–666. doi: 10.1111/VRU.12662, PMID: 29998490 PMC6218286

[ref8] Finno C. J. Miller A. D. Sisó S. Divers T. Gianino G. Barro M. V.. (2016). Concurrent equine degenerative myeloencephalopathy and equine motor neuron disease in three young horses. J. Vet. Intern. Med. 30, 1344–1350. doi: 10.1111/jvim.13977, PMID: 27298214 PMC5089576

[ref9] Garrett K. S. (2022). Special diagnostic techniques in equine neurology (radiography, ultrasonography, computed tomography, and magnetic resonance imaging). Vet. Clin. North Am. Equine Pract. 38, 171–188. doi: 10.1016/j.cveq.2022.04.001, PMID: 35810148

[ref10] Hahn C. N. Handel I. Green S. L. Bronsvoort M. B. Mayhew I. G. (2008). Assessment of the utility of using intra-and intervertebral minimum sagittal diameter ratios in the diagnosis of cervical vertebral malformation in horses. Vet. Radiol. Ultrasound 49, 1–6. doi: 10.1111/j.1740-8261.2007.00308.x, PMID: 18251286

[ref11] Hoey S. Stokes D. McAllister H. Puggioni A. Skelly C. (2022). A systematic review evaluating the use of ultrasound in the identification of osteochondrosis in horses. Vet. J. 282:105825. doi: 10.1016/j.tvjl.2022.105825, PMID: 35381440

[ref12] Hughes K. J. Laidlaw E. H. Reed S. M. Keen J. Abbott J. B. Trevail T.. (2014). Repeatability and intra-and inter-observer agreement of cervical vertebral sagittal diameter ratios in horses with neurological disease. J. Vet. Intern. Med. 28, 1860–1870. doi: 10.1111/JVIM.12431, PMID: 25410955 PMC4895627

[ref13] Janes J. G. Garrett K. S. Mcquerry K. J. Pease A. P. Williams N. M. Reed S. M.. (2014). Comparison of magnetic resonance imaging with standing cervical radiographs for evaluation of vertebral canal stenosis in equine cervical stenotic myelopathy. Equine Vet. J. 46, 681–686. doi: 10.1111/evj.12221 24329734

[ref14] Journée S. L. Journée H. L. de Bruijn C. M. Delesalle C. J. G. (2018). Multipulse transcranial electrical stimulation (TES): normative data for motor evoked potentials in healthy horses. BMC Vet. Res. 14, 121–129. doi: 10.1186/s12917-018-1447-7, PMID: 29615034 PMC5883272

[ref15] Levine J. M. J. M. Adam E. MacKay R. J. R. J. Walker M. A. Frederick J. D. Cohen N. D. (2007). Confirmed and presumptive cervical vertebral compressive myelopathy in older horses: a retrospective study (1992–2004). J. Vet. Intern. Med. 21, 812–819. doi: 10.1892/0891-6640(2007)21[812,capcvc]2.0.co;2 17708404

[ref16] Levine J. M. Scrivani P. V. Divers T. J. Furr M. Joe Mayhew I. Reed S.. (2010). Multicenter case-control study of signalment, diagnostic features, and outcome associated with cervical vertebral malformation-malarticulation in horses. J. Am. Vet. Med. Assoc. 237, 812–822. doi: 10.2460/JAVMA.237.7.812, PMID: 20919847

[ref17] Lindgren C. M. C. Wright L. Kristoffersen M. Puchalski S. M. (2020). Computed tomography and myelography of the equine cervical spine: 180 cases (2013–2018). Equine Vet. Educ. 33, 475–483. doi: 10.1111/eve.13350

[ref18] MacDonald D. B. Skinner S. Shils J. Yingling C. (2013). Intraoperative motor evoked potential monitoring - a position statement by the American Society of Neurophysiological Monitoring. Clin. Neurophysiol. 124, 2291–2316. doi: 10.1016/j.clinph.2013.07.025, PMID: 24055297

[ref19] Marks D. (1999). Cervical nerve root impingement in a horse, treated by epidural injection of corticosteroids. J. Equine Vet. 19, 399–401. doi: 10.1016/S0737-0806(99)80304-9

[ref20] Mayhew I. G. DeLahunta A. Whitlock R. H. Krook L. Tasker J. B. (1978). Spinal cord disease in the horse. Cornell Vet. 68, 1–207. 618720

[ref21] Mayhew I. G. Washbourne J. R. (1996). Magnetic motor evoked potentials in ponies. J. Vet. Intern. Med. 10, 326–329. doi: 10.1111/j.1939-1676.1996.tb02071.x, PMID: 8884720

[ref22] Nout Y. S. Reed S. M. (2003). Cervical vertebral stenotic myelopathy. Equine Vet. Educ. 15, 212–223. doi: 10.1111/j.2042-3292.2003.tb00246.x

[ref23] Pillai S. R. Traber M. G. Kayden H. J. Cox N. R. Toivio-Kinnucan M. Wright J. C.. (1994). Concomitant brainstem axonal dystrophy and necrotizing myopathy in vitamin E-deficient rats. J. Neurol. Sci. 123, 64–73. doi: 10.1016/0022-510x(94)90205-4, PMID: 8064324

[ref9001] Powers B. E. Stashak T. S. Nixon A. J. Yovich J. V. Norrdin R. W. (1986). Pathology of the vertebral column of horses with cervical static stenosis. Vet. Pathol. 23, 392–399. doi: 10.1177/030098588602300408 3750733

[ref24] Ricardi G. Dyson S. J. (1993). Forelimb lameness associated with radiographic abnormalities of the cervical vertebrae. Equine Vet. J. 25, 422–426. doi: 10.1111/J.2042-3306.1993.TB02984.X 8223374

[ref25] Rijckaert J. Pardon B. Saey V. Raes E. Van Ham L. Ducatelle R.. (2019). Determination of magnetic motor evoked potential latency time cutoff values for detection of spinal cord dysfunction in horses. J. Vet. Intern. Med. 33, 2312–2318. doi: 10.1111/JVIM.15576, PMID: 31490026 PMC6766509

[ref26] Rovel T. Zimmerman M. Duchateau L. Adriaensen E. Mariën T. Saunders J. H.. (2021). Computed tomographic myelography for assessment of the cervical spinal cord in ataxic warmblood horses: 26 cases (2015–2017). J. Am. Vet. Med. Assoc. 259, 1188–1195. doi: 10.2460/javma.20.11.0614, PMID: 34727080

[ref27] Scrivani P. V. Levine J. M. Holmes N. L. Furr M. Divers T. J. Cohen N. D. (2011). Observer agreement study of cervical-vertebral ratios in horses. Equine Vet. J. 43, 399–403. doi: 10.1111/J.2042-3306.2010.00300.X, PMID: 21496073

[ref28] Sleutjens J. Cooley A. J. Sampson S. N. Wijnberg I. D. Back W. van der Kolk J. H.. (2014). The equine cervical spine: comparing MRI and contrast-enhanced CT images with anatomic slices in the sagittal, dorsal, and transverse plane. Vet. Q. 34, 74–84. doi: 10.1080/01652176.2014.951129 25174534

[ref29] Summers B. A. Cummings J. F. DeLahunta A. (1995). Veterinary neuropathology. Available at: https://www.worldcat.org/nl/title/30623436.

[ref30] Van Biervliet J. (2007). An evidence-based approach to clinical questions in the practice of equine neurology. Vet. Clin. North Am. Equine Pract. 23, 317–328. doi: 10.1016/j.cveq.2007.03.009, PMID: 17616316

[ref31] Van Biervliet J. Scrivani P. V. Divers T. J. Erb H. N. de Lahunta A. Nixon A. (2004). Evaluation of decision criteria for detection of spinal cord compression based on cervical myelography in horses: 38 cases (1981-2001). Equine Vet. J. 36, 14–20. doi: 10.2746/0425164044864642, PMID: 14756366

[ref9002] Wagner P. C. Grant B. D. Reed S. M. (1987). Cervical vertebral malformations. Vet. Clin. North Am. Equine Pract. 3, 385–396. doi: 10.1016/S0749-0739(17)30681-8 3304571

[ref32] Yovich J. V. LeCouteur R. A. Gould D. H. (1991). Chronic cervical compressive myelopathy in horses: clinical correlations with spinal cord alterations. Aust. Vet. J. 68, 326–334. doi: 10.1111/j.1751-0813.1991.tb03091.x, PMID: 1755784

